# Genomic Analysis of Natural Selection and Phenotypic Variation in High-Altitude Mongolians

**DOI:** 10.1371/journal.pgen.1003634

**Published:** 2013-07-18

**Authors:** Jinchuan Xing, Tana Wuren, Tatum S. Simonson, W. Scott Watkins, David J. Witherspoon, Wilfred Wu, Ga Qin, Chad D. Huff, Lynn B. Jorde, Ri-Li Ge

**Affiliations:** 1 Eccles Institute of Human Genetics, University of Utah School of Medicine, Salt Lake City, Utah, United States of America; 2 Research Center for High Altitude Medicine, Qinghai University Medical School, Xining, Qinghai, People's Republic of China; Dartmouth College, United States of America

## Abstract

Deedu (DU) Mongolians, who migrated from the Mongolian steppes to the Qinghai-Tibetan Plateau approximately 500 years ago, are challenged by environmental conditions similar to native Tibetan highlanders. Identification of adaptive genetic factors in this population could provide insight into coordinated physiological responses to this environment. Here we examine genomic and phenotypic variation in this unique population and present the first complete analysis of a Mongolian whole-genome sequence. High-density SNP array data demonstrate that DU Mongolians share genetic ancestry with other Mongolian as well as Tibetan populations, specifically in genomic regions related with adaptation to high altitude. Several selection candidate genes identified in DU Mongolians are shared with other Asian groups (*e.g.*, *EDAR*), neighboring Tibetan populations (including high-altitude candidates *EPAS1*, *PKLR*, and *CYP2E1*), as well as genes previously hypothesized to be associated with metabolic adaptation (*e.g.*, *PPARG*). Hemoglobin concentration, a trait associated with high-altitude adaptation in Tibetans, is at an intermediate level in DU Mongolians compared to Tibetans and Han Chinese at comparable altitude. Whole-genome sequence from a DU Mongolian (Tianjiao1) shows that about 2% of the genomic variants, including more than 300 protein-coding changes, are specific to this individual. Our analyses of DU Mongolians and the first Mongolian genome provide valuable insight into genetic adaptation to extreme environments.

## Author Summary

Throughout history, Mongolians have survived the harsh conditions of northern latitudes, including seasonal cold, drought, and a restricted diet. Approximately 500 years ago, nomadic Deedu (DU; “at high altitude”) Mongolians migrated from the Mongolian steppes to the northeastern highlands of the Qinghai-Tibetan Plateau. Using high-density SNP data, we demonstrate that present-day DU Mongolians share genetic ancestry with other Mongolians and with Tibetans. High-altitude selection candidate genes previously identified in the latter population (*EPAS1*, *PKLR*, *CYP2E1*), and *PPARG*, a gene long hypothesized to play a role in metabolic adaptation, are among the strongest adaptive signals in DU Mongolians. Furthermore, we show that hemoglobin concentration, associated with high-altitude adaptation in Tibetans, is intermediate in DU Mongolians compared to Tibetans and Han Chinese at comparable altitudes. Whole-genome sequence from a DU Mongolian shows that approximately 300 protein-coding changes are specific to this individual. Our analyses provide new perspectives on genetic variation and adaptation to extreme environments.

## Introduction

Prehistoric Mongolian ancestry can be traced to the Gobi and Mongolian steppes in Northeastern Asia, yet Mongolian lineages are found among present-day inhabitants of regions as far west as Eastern Europe [1]–[3]. This vast genetic signature is largely attributed to population movements during the time of Genghis Khan [3], [4], whose efforts to unite Eurasian tribes during the 13^th^ century helped to establish one of the largest contiguous empires in human history (12 million square miles) [5], [6]. Assimilation with resident inhabitants throughout this vast and varied region has resulted in a complex mixture of genetic variation and cultural practices in Mongolians that may promote survival in novel environments [7], [8]. A better understanding of Mongolian genetic diversity will provide important insights into genetic variation throughout northern Eurasia and genetic adaptation in highly variable environments.

Through much of their history, Mongolians have survived the harsh conditions of northern latitudes, including seasonal cold and drought and a highly restricted diet, and they might have genetically adapted to these conditions. More than 500 years ago, the nomadic Deedu (“at high altitude”) Mongolians (referred to as “DU Mongolians” hereafter) migrated from the Mongol steppes to the northeastern section of the Qinghai-Tibetan Plateau [5], [6], [9]. In this new environment (∼3000 meters above sea level), they have been further challenged by hypoxic conditions. Adaptations to the challenge imposed by hypoxia have been studied in highland Tibetan populations, and putatively adaptive genetic factors that are associated with both hypoxia and metabolic factors have been reported [10]. In addition, adaptations to hypoxia and cold have also been reported in high-altitude deer mice, highlighting regulatory shifts in energy utilization and thermogenic capacity [11]. In humans, putatively adaptive genetic factors in highland Tibetans are also associated with metabolic factors that may be linked with cold tolerance [10]. While it is unlikely that genome-wide selection analyses would identify selective events that occurred since their arrival into the Qinghai-Tibetan Plateau about 25 generations ago, we hypothesize that shared ancestry and recent genetic admixture from neighboring long-term highland residents play a crucial role in the adaptation of DU Mongolians to this region.

By examining genome-wide SNPs in a DU Mongolian population and whole-genome sequence data from a DU Mongolian individual, we are able to explore Mongolian genetic variation at an unprecedented level of detail. Our analysis identifies hypoxia, antimicrobial, and metabolic selection candidates and reveals novel variation in the genomes of this unique population.

## Results

### Population Genetic Analysis

**Figure 1:**
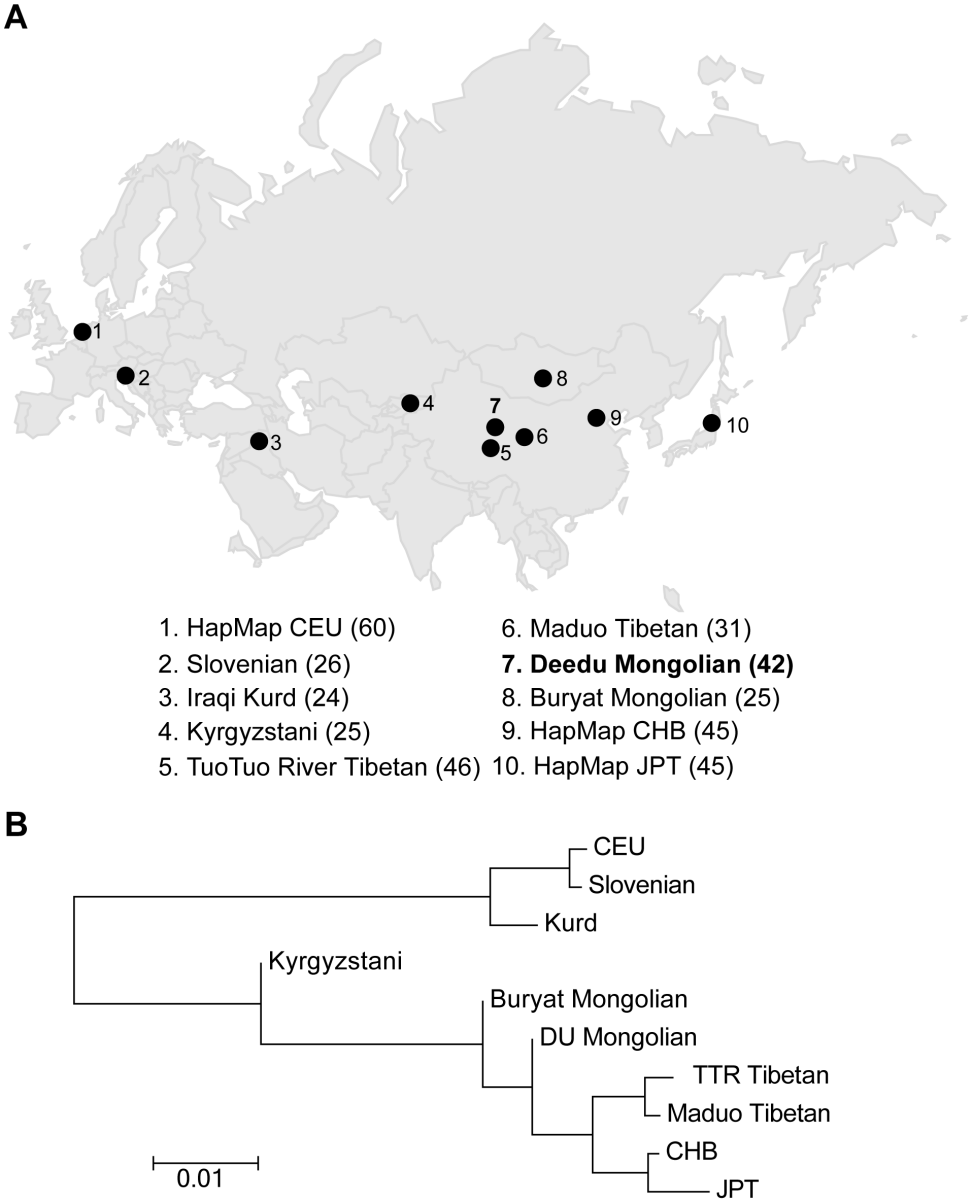
A) Northern Eurasian populations analyzed. The sampling location of each population is shown on the map, and the number of individuals in each population is shown at the bottom of the figure. B) Neighbor-joining tree for northern Eurasian populations based on the pairwise *F_ST_*.

To assess the population structure of DU Mongolians and their relationship with surrounding populations, we examined ∼860,000 SNPs genotyped in 369 individuals from 10 Eurasian populations (Figure 1A). A pairwise *F_ST_* analysis (Table 1) indicates the DU Mongolians are most closely related to the Buryat Mongolians (*F_ST_* = 0.0075), followed by the Kyrgyzstani population and the Tibetan population from the Tuo Tuo River (TTR) area of the Qinghai-Tibetan Plateau (*F_ST_* = 0.0105 and 0.0114, respectively). A neighbor-joining tree from the pairwise *F_ST_* values showed similar patterns, with DU Mongolians positioned outside of the clade that includes Tibetans, Han Chinese and Japanese (Figure 1B).

**Table 1:** Pairwise *F_ST_* among Eurasian populations.

	DU Mongolian	Buryat Mongolian	Kyrgyzstani	TTR Tibetan	Maduo Tibetan	CHB	JPT	Kurd	Slovenian
Buryat Mongolian	**0.008**								
Kyrgyzstani	**0.011**	0.008							
TTR Tibetan	**0.011**	0.020	0.024						
Maduo Tibetan	**0.013**	0.017	0.022	0.004					
CHB	**0.013**	0.016	0.023	0.014	0.012				
JPT	**0.016**	0.017	0.025	0.020	0.018	0.007			
Kurd	**0.081**	0.081	0.047	0.102	0.099	0.106	0.108		
Slovenian	**0.085**	0.085	0.050	0.107	0.104	0.111	0.113	0.013	
CEU	**0.086**	0.085	0.051	0.107	0.104	0.111	0.113	0.014	0.003

**Figure 2:**
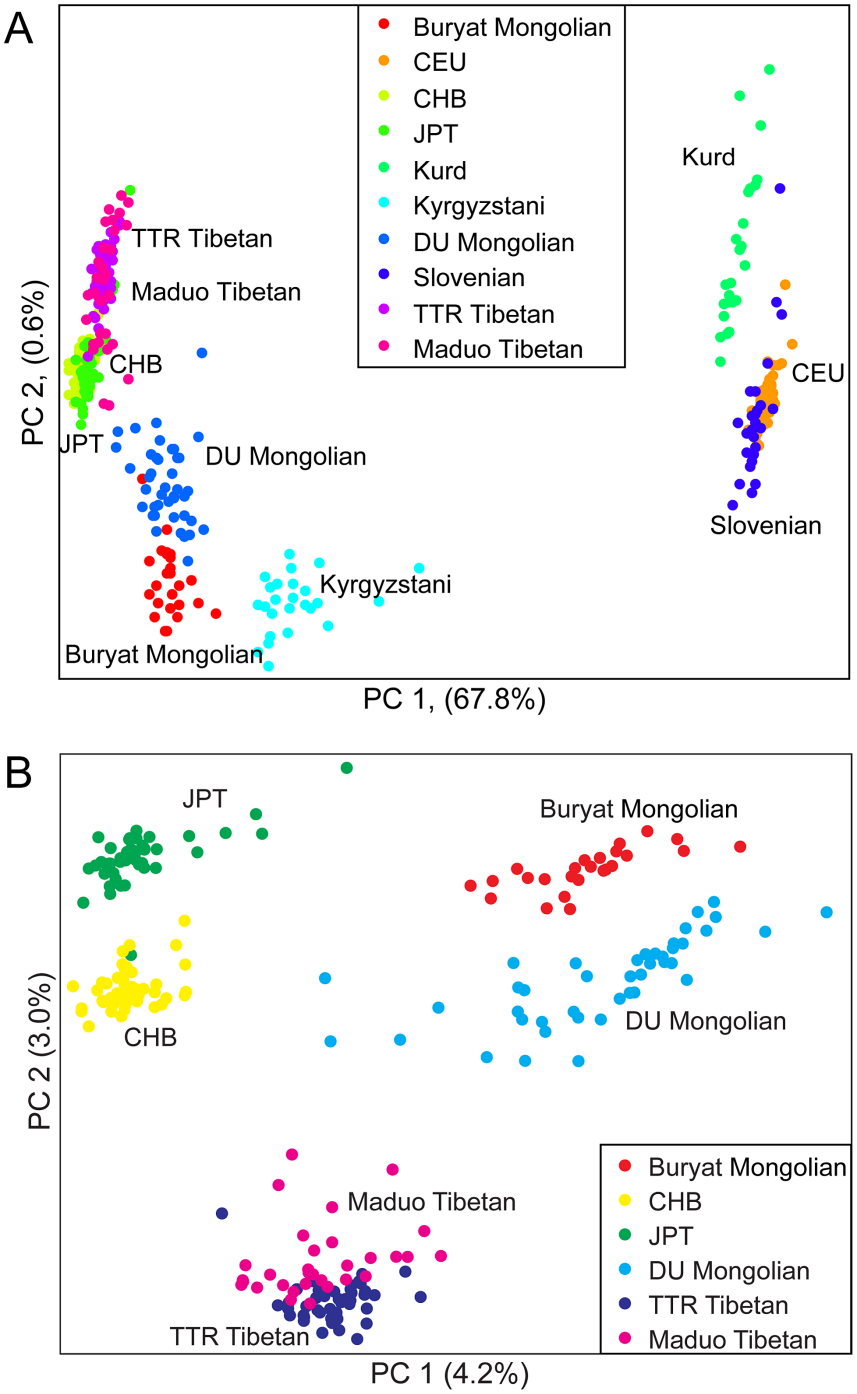
Principal components analysis of A) ten northern Eurasian populations; and B) six East Asian populations. First two principal components are shown. Each individual is represented by one dot and the color label corresponding to their population. The percentage of variance explained by each PC is shown on the axis.

To further examine the relationships among individuals, we performed principal components analysis (PCA) using pairwise genetic distances among all individuals. Two-thirds (67.8%) of the variance in the dataset can be explained by the first principal component (PC1), separating populations from Europe and West Asia from populations from East and Central Asia (Figure 2A). PC2 only accounts for <1% of the variance, separating Mongolians, Tibetans, HapMap CHB and JPT individuals, and Kyrgyzstanis. When only East Asian populations were examined, DU Mongolians are most closely related to Buryat Mongolians (Figure 2B). Two Tibetan populations (from the TTR and Maduo regions), HapMap CHB, and HapMap JPT are all clearly separated from the Mongolian populations. Although the majority of the DU Mongolian individuals form a relatively distinct cluster, several individuals show more similarity to other East Asian populations, such as Tibetans and Han Chinese, suggesting possible gene flow among these populations.

**Figure 3:**
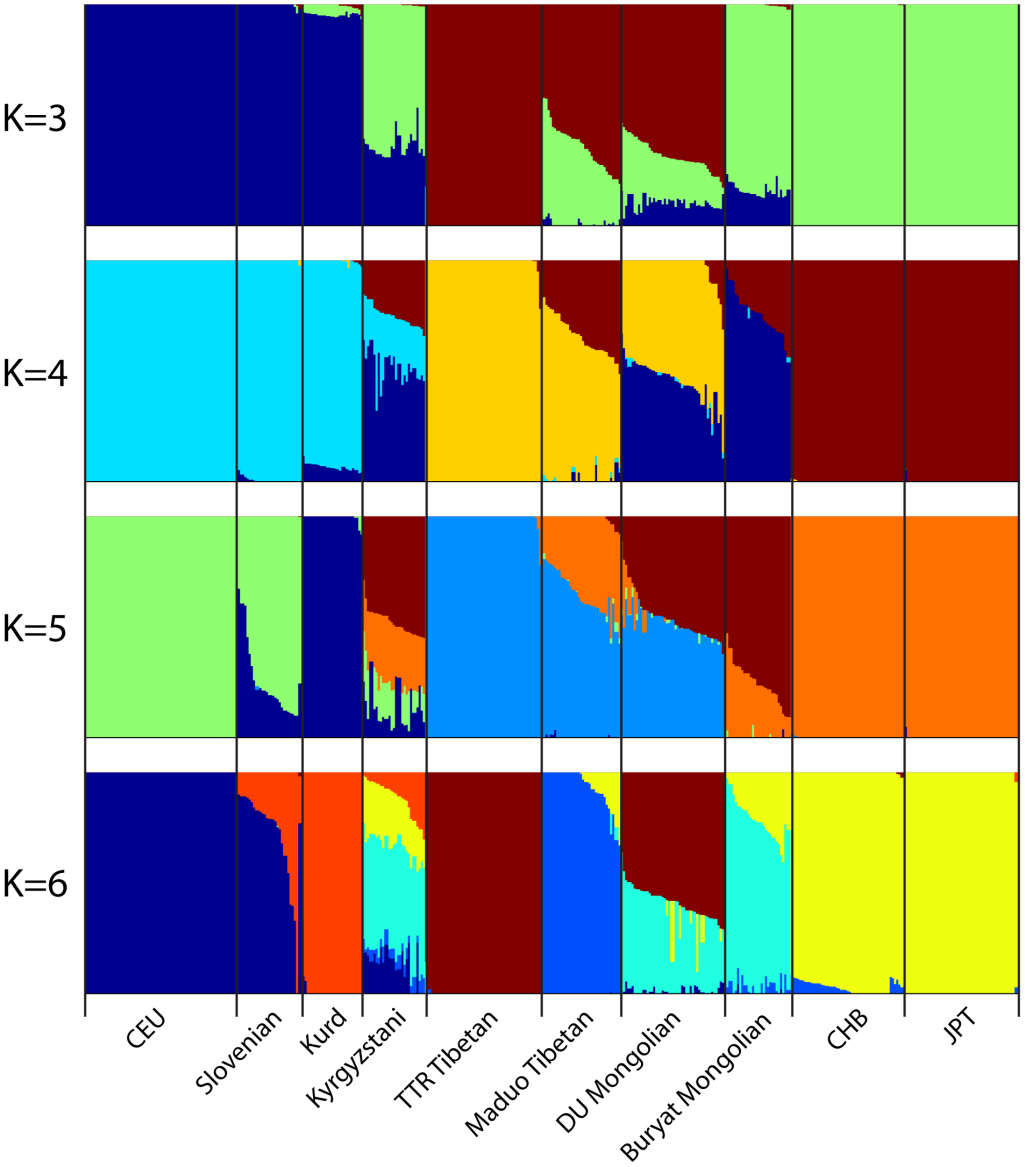
Genome-wide admixture of Eurasian individuals inferred by *ADMIXTURE*. Results from K = 3 to K = 6 are shown. Each individual's genome is represented by a vertical bar composed of colored sections, where each section represents the proportion of an individual's ancestry derived from one of the K ancestral populations. Individuals are arrayed horizontally and grouped by population as indicated.

Next we used the program *ADMIXTURE* to assess the genetic composition of DU Mongolians and neighboring populations. The *ADMIXTURE* analysis estimates the ancestry of each individual, assuming 3–6 ancestral populations (K) (Figure 3). At K = 3, ancestral components corresponding to European/West Asian, Tibetan/DU Mongolian, and East Asian/Buryat Mongolians are recognized. At K = 4, a Mongolian component including DU and Buryat Mongolians is recognized. DU Mongolian genomes appear to be mixtures of ancestral Mongolian and Tibetan components. These two components are closely related and separate from other East Asians (HapMap CHB/JPT). On average, DU Mongolian genomes are composed of 44% of the Mongolian component (standard deviation [sd] 11%), 52% of the Tibetan components (sd 6%), and 3% East Asian component (sd 8%). In contrast, the Buryat Mongolian genomes are composed primarily of Mongolian (75%, sd 10%) and East Asian (25%, sd 9%) components. This result suggests that unlike Buryat Mongolians, DU Mongolians share substantial genetic components with Tibetans, either due to recent admixture or more ancient shared ancestry. At K = 6, Maduo and TTR Tibetans were recognized as two separate groups, and DU Mongolian genomes appear as mixtures of TTR Tibetan and Mongolian components. Some of the DU Mongolian individuals also contain more than 5% East Asian components. The Kyrgyzstanis from Central Asia have the most genetic admixture, and about half of their ancestry is accounted by the ancestral Mongolian component at K = 4 to 6.

### Selection Analysis

We performed iHS and XP-EHH analyses to identify candidate regions targeted by positive selection in the DU and Buryat Mongolian populations. In total, 96 regions (68 containing genes; 28 non-genic regions) comprising 162 candidate genes were identified in both DU and Buryat Mongolian populations in at least one of the two selection analyses (Table S1).

We hypothesize that selection candidates identified in both Mongolian populations would be associated with factors common to ancestral Mongolian groups at northern latitudes (see [12] for review) such as metabolic adaptations to temperature and diet. Indeed, a number of metabolic genes are identified in the overlapping candidate gene sets (*e.g.*, *ADRA2A*, *MYOF*, and *CYP26A1/C1*). In addition, we identified a gene cluster containing six beta-defensin genes (*DEFB125*, *DEFB126*, *DEFB127*, *DEFB128*, *DEFB129*, and *DEFB132*) among the selection candidates. It is known that genes related to immune system and microbial defense are highly variable [13]. In particular, this defensin gene cluster varies in copy number in humans and might have undergone copy number expansion in East Asian populations [14].

Considering the geographic proximity of Mongolians and Tibetans, as well as their shared cultural practices and environmental conditions, we also examined the overlap of selection targets among these populations. A total of 153 selection regions containing 399 genes were identified as significant (p<0.02) in at least one Mongolian and one Tibetan population (Table S2). Among the top selection candidates is *PPARG* (peroxisome proliferator-activated receptor gamma), which has been hypothesized as a “thrifty” gene for dietary adaptation [15], [16]. *PPARG* may also be associated with metabolism-related traits involving cold tolerance and energy utilization, such as thermogenesis and brown adipose tissue [17]. A number of selection candidate genes reported in previous studies of Asian populations were also found in the Mongolian-Tibetan overlapping candidates. For example, *EDAR* (the ectodysplasin A receptor gene is significant in both the DU Mongolians and the TTR Tibetans (iHS, p = 0.004 and 0.016, respectively). *EDAR* is involved in the development of hair, teeth, and exocrine glands [7] and has been highlighted as a strong selection candidate in several Asian populations [18], [19].

A total of six regions (15 total candidate genes) identified in our analyses are significant in all four Mongolian and Tibetan populations (Table S2). The number of shared regions is significantly higher than the expected number (∼0.3, p = 0) under a null hypothesis of independence. This highly refined list of selection candidates, which contains genes related to muscle metabolism and angiogenesis (*MYOF*, *MAP2K5*) and tumor suppression (*MCC*), yields valuable insight for future examination of adaptation in both Mongolian and Tibetan ethnic groups.

**Figure 4:**
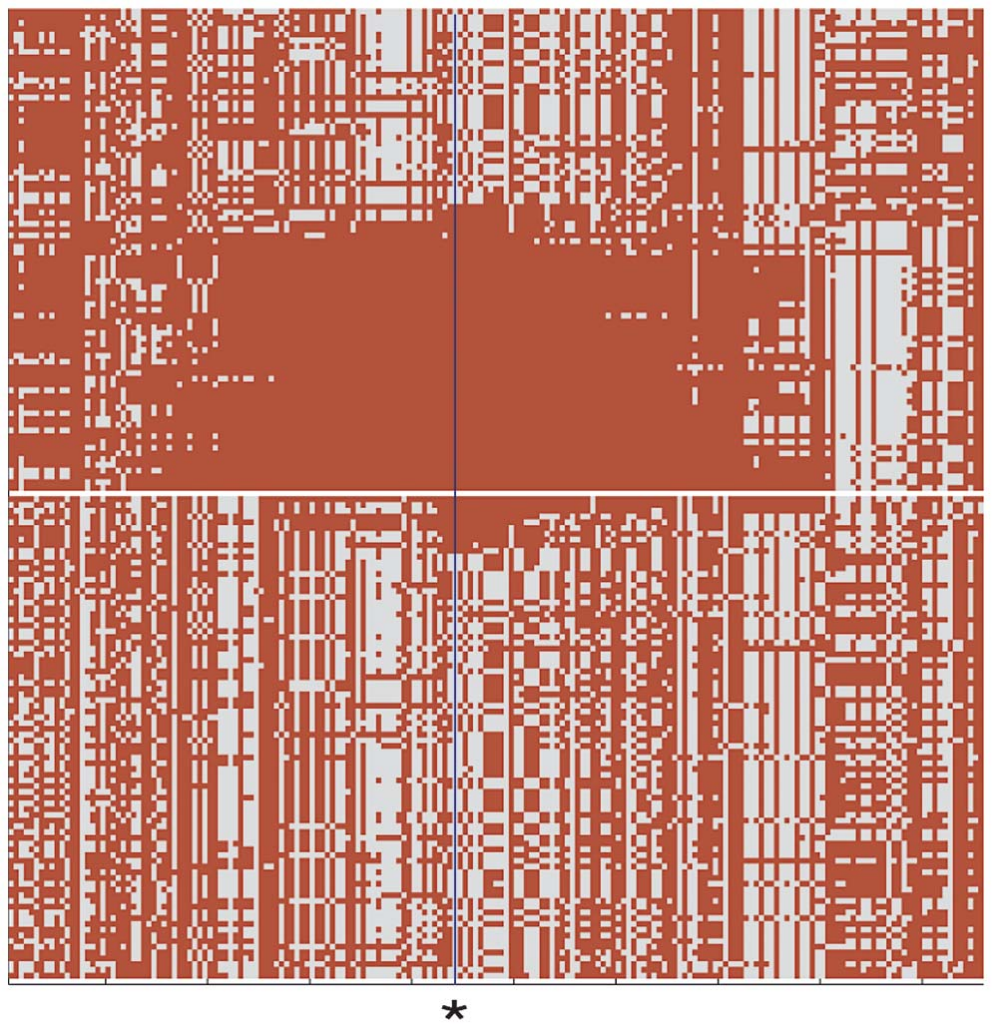
Comparison of DU Mongolian and HapMap CHB haplotypes at the *EPAS1* genomic region. Haplotype pattern at the *EPAS1* genomic region in the 84 chromosomes of 42 DU Mongolian individuals and 84 randomly drawn chromosomes from 45 CHB individuals is shown. An asterisk at position 46490868 on chromosome 2 indicates the core SNP haplotype for the *EPAS1* genomic region shown (chr2:46304028–46851921; 191 SNPs total); all haplotypes were sorted as previously described (Figure 1 of [20]).

We next compared putatively selected regions (p<0.02) in DU Mongolians with genes that have been previously associated with high-altitude adaptation in Tibetans [20]–[25]. Three Tibetan high-altitude selection candidates are also highly significant in DU Mongolians, including the hypoxia inducible pathway gene *EPAS1* [20]–[22], [24]–[27] (Figure 4); the *PKLR* gene [20]–[22], which encodes liver- and erythrocyte-expressed pyruvate kinase; and the cytochrome P450 family member *CYP2E1* [20]. These hypoxia-related genes are also associated with metabolic processes [17], [23] that may be involved in orchestrating important physiological responses at high altitude. To control for the effect of admixture among the DU Mongolians, we also performed two SNP-based selection scans in addition to the haplotype based selection analysis. Results from the population branch statistic (PBS) and multiple regression analysis (MR) are shown in Tables S3 and S4, respectively. Among the high-altitude candidate genes examined, *EPAS1* showed significant signal (p<0.005) in the PBS analysis. *PPARG*, a selection candidate identified among the top 2% of iHS selection candidates in Tibetans and DU Mongolians, is also significant in the DU Mongolian PBS analysis (p<0.005).

### Hemoglobin Concentration in DU Mongolians

Before DU Mongolians migrated to the Qinghai-Tibetan plateau, Tibetans had lived on the plateau for thousands of years and had adapted genetically to high altitude [28], [29]. On average, Tibetans exhibit relatively lower hemoglobin concentration ([Hb]) compared to other groups at comparable high altitude [30], [31], and this unique characteristic is associated with adaptive genetic factors [20]–[22].

The *ADMIXTURE* results support shared ancestry and possible gene flow from Tibetans to DU Mongolians (Figure 3), which in turn suggests the hypothesis that Tibetan high-altitude adaptive genes have been transferred to the DU Mongolian population. To test this hypothesis, we measured [Hb] in DU Mongolians and compared the results to those of Tibetans and Han Chinese from a previous study [32] (Table 2). The [Hb] of the Tibetan and Han Chinese samples were collected at an altitude (2664+/−258 m), comparable to the region where the DU Mongolian samples were collected (∼3000 m). When the individuals are divided into two age groups, the average [Hb] of DU Mongolian females is similar to Tibetans and significantly lower than Han Chinese in the 16–40 year age group (p<0.006), but is similar to Han Chinese and significantly higher than Tibetans in the 41–60 year age group (p<0.007). Previously reported haplotypes that have undergone selection in Tibetans [20] are not associated with [Hb] in this sample (Table S5).

**Table 2:** [Hb] comparisons among DU Mongolians, Tibetans, and Han Chinese.

	Age 16–40				Age 41–60				Age 16–60			
	[Hb] (g/dL)	Std	N	p^a^	[Hb] (g/dL)	Std	N	p^a^	[Hb] (g/dL)	Std	N	p^a^
DU Mongolian	12.6	1.2	10		14.4	1.2	10		13.5	1.5	20	
Tibetan	12.8	1.5	150	0.62	13	1.6	176	**0.007**	12.9	1.6	326	0.10
Han Chinese	14	1.7	174	**0.006**	14.5	1.5	161	0.87	14.2	1.6	335	**0.049**

a p value of the t-test comparing [Hb] between DU Mongolian and Tibetan/Han Chinese.

### Whole-Genome Sequence of a DU Mongolian

To identify potential functional variants in DU Mongolians, and to compare patterns of genetic variation from whole-genome sequencing with those based on SNP microarrays, we performed whole-genome sequencing on a DU Mongolian male (Tianjiao1) who was born and raised on the Qinghai-Tibetan Plateau. Relative to the reference sequence, a total of 3,803,076 variants were identified in Tianjiao1, including 3,353,824 single nucleotide variations (SNVs), 198,230 deletions, 180,121 insertions (a total of 378,351 indels), and 70,901 complex substitutions and multiple nucleotide polymorphisms (Table 3). In addition, 170 copy number variations (CNV) and 1439 high-confidence structural variants (SV) were identified.

**Table 3:** Summary of variants in the Tianjiao1 genome.

	Total	Not in CGI54	%	Not in CGI54/1000 Genomes	%
SNV	3,353,824	92,310	2.75%	44,783	1.34%
Insertion	180,121	16,992	9.43%	16,435	9.12%
Deletion	198,230	14,896	7.51%	11,886	6.00%
Substitution^a^	70,901	3,830	5.40%	2,444	3.45%
Total	3,803,076	128,028	3.37%	75,548	1.99%

a Including complex substitutions and multi-nucleotide polymorphisms.

Using whole-genome data, we determined the mitochondrial and Y haplogroups of Tianjiao1 (Table S6). The Tianjiao1 mtDNA sequence is assigned to clade H18 [33], which has been previously reported in Europeans [34], [35] and is also present in the Arabian Peninsula and Caucasus regions. H18 is estimated to have originated ∼13,500 years ago [36]. The Tianjiao1 Y chromosome produced 1,897 variant calls and can be assigned to haplogroup Q1a1 based on the current Y-DNA haplotype tree [37]. The Q1a1 (M120) Y-chromosome lineage has been reported previously at low frequencies in populations from Mongolia, central and northern Asia, the Lhasa region of Tibet, and in Han Chinese [38], [39].

**Figure 5:**
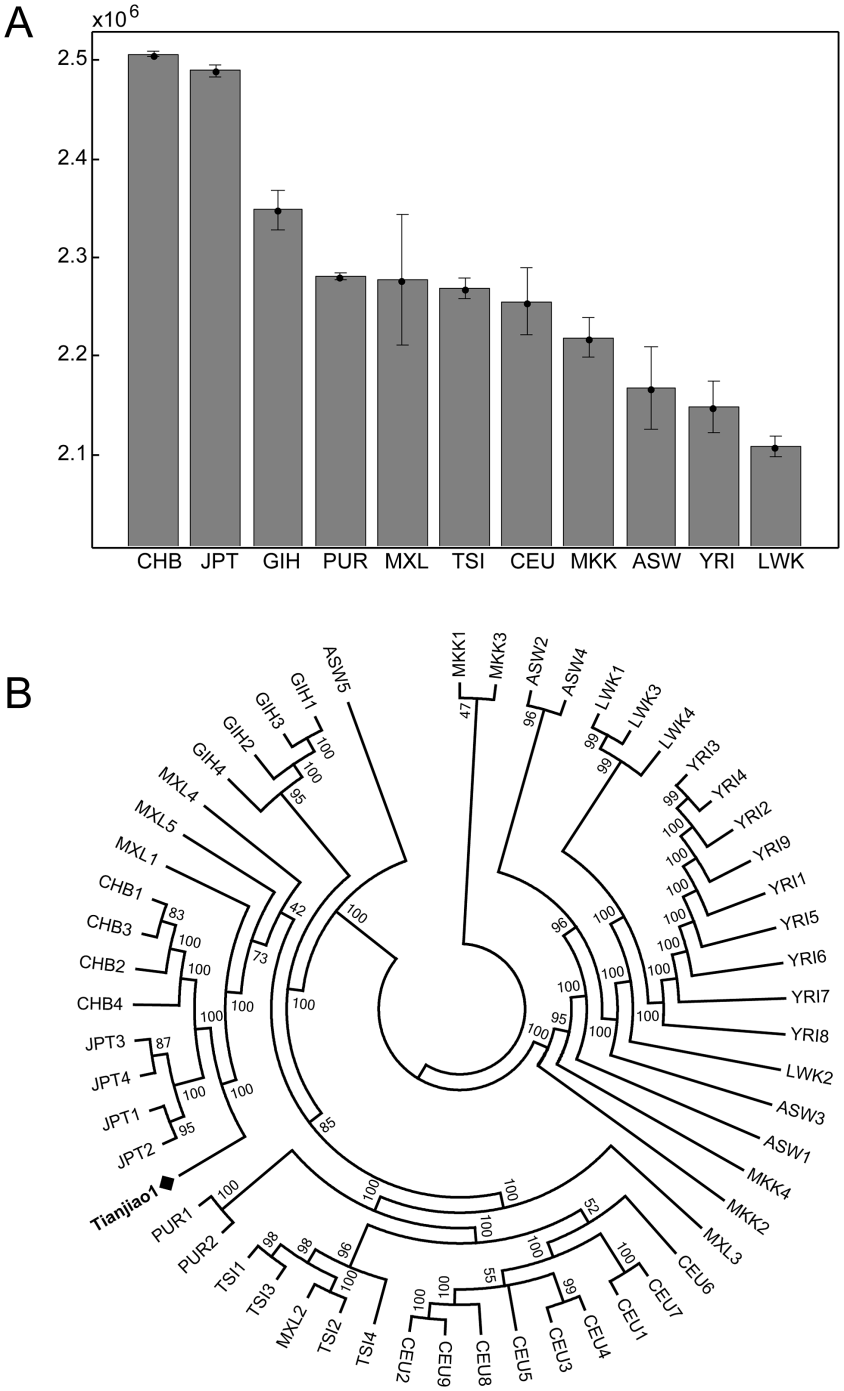
A) Number of variants shared between Tianjiao1 and CGI54 panel individuals. CGI54 panel individuals were grouped by populations, and the mean number of shared variants and standard deviation for each population are shown. Population code: CHB: Han Chinese; JPT: Japanese; GIH: Gujarati; PUR: Puerto Rican; MXL: Mexican-American; TSI: Tuscan; CEU: Utah residents (CEPH) with Northern and Western European ancestry; MKK: Maasai; ASW: African-American; YRI: Yoruba; LWK: Luhya. B) Neighbor-joining tree of Tianjiao1 and the CGI54 panel individuals. The bootstrap value for each branch is shown.

We compared the Tianjiao1 genome to whole genome sequences of 54 unrelated individuals sequenced using the same sequencing platform and variant discovery pipeline. These 54 individuals (referred to as “CGI54 panel” hereafter) were selected from 11 populations across the world and are a good representation of overall genomic diversity in humans. The total number of variants in the Tianjiao1 genome is similar to that of other Eurasian individuals and is lower than African individuals in the CGI54 panel (Figure S1). Tianjiao1 shared most variants with CHB individuals and least with LWK (Luhya in Webuye, Kenya) individuals (Figure 5A). A neighbor-joining tree based on pairwise genetic distances demonstrates the affinity of Tianjiao1 to other East Asian individuals (Figure 5B). This whole-genome comparison result is consistent with our PCA results (Figure 2). In our *ADMIXTURE* analysis (K = 4), the Tianjiao1 genome appears to be a mixture of three ancestral components and is estimated to contain 26%, 56%, and 17% Mongolian, Tibetan, and HapMap CHB/JPT ancestry, respectively.

### Novel Variants with Potential Functional Impact

To assess the functional impact of variants in this sequence, we annotated the effects of the variants using the terms defined in Sequence Ontology [40]. The number of variants in each category is shown in Table 4. We found there are 1,467,962 genic variants, including 19,964 coding sequence variants. Among the coding variants, 9,216 are non-synonymous substitutions. There are also 62 stop-gain and 28 stop-lost SNVs. Among indels, 160 are in-frame and 134 are frame-shifting. Within introns, 61 mutations are located at the splice donor sites and 45 are located at splice acceptor sites (Table 4). We compared these functional variants with the Human Gene Mutation Database (HGMD) to identify variants that are previously known to be associated with disease phenotypes. A total of 43 HGMD mutations are present in the Tianjiao1 genome, including 9 homozygous autosomal mutations and one hemizygous mutation on chromosome X. All but one of these variants are known SNVs that are present in samples from the 1000 Genomes project. The only nonsense mutation (chr17:15142830C->G) that is specific to Tianjiao1 is located in the *PMP22* gene (peripheral myelin protein 22 [HGNC 9118]) and has been associated with Charcot-Marie-Tooth disease (CMT1A, [MIM 118220]) [41]. Previous studies of DNA sequences from apparently healthy individuals have yielded similar results [42]–[45].

**Table 4:** Summary of genic variants in the Tianjiao1 genome.

	Total	Not in CGI54	Not in CGI54/1000 Genomes
**Intron**	1,416,818	51,164	30,024
**splice acceptor**	45	3	3
**splice donor**	61	2	2
**Exon**	57,031	2,391	1,290
**5′ UTR**	3,964	141	84
**3′ UTR**	26,853	1,079	638
**other non-coding**	8,625	296	177
**synonymous**	10,386	380	139
**non-synonymous**	9,216	540	255
**frame-shift**	134	36	34
**in-frame**	160	21	17
**stop gain**	62	8	5
**stop lost**	28	0	0
**stop retain**	4	0	0
**Total Genic**	1,467,962	53,354	31,216

A comparison of the variants found in Tianjiao1 with those of the CGI54 panel and the 1000 Genomes Project shows that 31,216 variants are specific to Tianjiao1. Of these, 449 are present in the coding regions, including 139 synonymous SNVs, 255 non-synonymous SNVs, 5 nonsense SNVs, 34 frame-shifting indels, and 17 in-frame indels (Table 4). Using the program SIFT [46], we predicted the functional impact of non-synonymous SNVs and coding indels specific to Tianjiao1. In addition to the 5 stop-gain SNVs, 77 non-synonymous SNVs were predicted to be “Damaging” or “Possibly damaging” (Table S7). Among the 51 coding indels, 8 are within the first 10% of the transcript and 23 were predicted to cause nonsense-mediated decay of the transcript (Table S8).

### Coding Variants in the Candidate Regions Under Natural Selection

Finally, we examined Tianjiao1-specific coding variants in the putatively selected regions that are shared by Mongolians (Table S1) and between Mongolians and Tibetans (Table S2). Among all regions, two Tianjiao1-specific heterozygous nonsynonymous SNVs were identified in genes *PAOX* and *EPHB6*, respectively. The mutation in the *PAOX* gene (p.D274G) was predicted to be “Damaging” while the mutation in *EPHB6* (R413L) was predicted to be “Tolerated” by SIFT (Table S7). Within these regions, no coding indels, CNVs, or SVs were identified in the Tianjiao1 genome.

## Discussion

Our results show that DU Mongolians form a distinct population compared to other Eurasian populations and, among our samples, have closest genetic similarity to Buryat Mongolians. The *ADMIXTURE* analysis suggests that DU Mongolians share appreciable amounts of ancestry with neighboring Tibetan populations. This ancestral component is distinct from other East Asian populations. Genetic and archaeological evidence indicates that regions of the southern portion of the Tibetan Plateau were first occupied during the Late Pleistocene (∼30,000 years ago) [25], [47], [48]. Approximately 3,750–6,500 years ago, additional populations migrated from the east into the northeastern section of present-day Qinghai Province and the easternmost regions of Tibet [47]. The ancestry shared among Qinghai Mongolian and Tibetan populations examined here may be attributed to the latter more recent migration into the northeastern section of the Qinghai-Tibetan Plateau.

Because of the shared ancestry and possible gene flow between DU Mongolians and Tibetans, one intriguing question is whether high-altitude adaptive alleles have been transferred to the DU Mongolians after their migration to the Qinghai-Tibetan plateau. DU Mongolians specifically exhibit strong signals of selection for genes previously reported as candidates for high-altitude adaptation in neighboring Tibetan populations, including *EPAS1*, *PKLR*, and *CYP2E1* (p<0.02 in DU Mongolian and Tibetan populations). The endothelin receptor type A (*EDNRA*) selection candidate gene reported by Simonson et al. [20] is significant in Buryat but not DU Mongolians, suggesting an adaptive role that may not be strictly hypoxia-specific.

Our comparison of selection candidate genes among Buryat and DU Mongolians and two Tibetan populations yields candidate genes that may be related to metabolic factors involved in adaptation to cold, arid conditions and a relatively restricted diet (Table S2). Notably, the *PPARG* gene, previously hypothesized to be associated with metabolic adaptation in human populations [12], [15], [16], exhibits a strong signal of selection in Mongolian and Tibetan populations and warrants further investigation. The individual variant-based selection scans, PBS and MR, showed some overlap in selection candidate regions with our haplotype based scan, although the top regions are largely different. For example, the *EPAS1* gene was identified as a strong selection candidate gene in DU Mongolians by the XP-EHH and PBS tests but not in the iHS and MR tests, reflecting differences in these approaches.

Because of the shared recent ancestry of these populations, it is unclear if these selection targets are the outcome of more ancient selective events common to ancestors of these groups or whether Mongolians and Tibetans exchanged favorable genes in more recent history. While we did not detect an association between putative selected Tibetan haplotypes and [Hb] in DU Mongolians, the difference between DU Mongolian and Han Chinese [Hb] phenotypes at an altitude of 3000 m suggests that DU Mongolians might share Tibetans' phenotype of decreased [Hb] in a hypoxic environment.

Our whole-genome sequence and genome-wide SNP analyses provide the first genomic-level insight into a Mongolian population, augmenting our current understanding of human genetic variation. Further studies of this unique population will elucidate the evolutionary underpinnings of adaptive signals shared among other Mongolian populations in addition to neighboring Tibetan groups. Efforts focused on Mongolian genomics beyond the single genome analysis presented here will also provide greater insight into direct targets of selection that are likely associated with important physiological and metabolic traits.

## Materials and Methods

### Sample Collection

Blood samples from all DU Mongolian subjects were drawn at an altitude of 3000 meters (m). All the subjects are nomads who permanently live at an altitude of 3000 m–4300 m in the Qinghai Keke Xili area. DNA was extracted from whole blood samples using the Qiagen Gentra Puregene Blood Kit (Qiagen Inc., Valencia, California, USA). Informed consent was obtained for all participants according to guidelines approved by the Institutional Review Board at the High Altitude Medical Research Institute, Qinghai Medical College (Xining, Qinghai, China).

A healthy, 58-year-old DU Mongolian male who was born and raised at an altitude of 3250 m on the Qinghai-Tibetan Plateau provided a blood sample from which DNA was extracted for whole-genome sequencing. The sample collection was carried out in Salt Lake City, Utah, and whole-genome sequencing was undertaken at Complete Genomics, Inc. Both procedures were performed with Institutional Review Board approval under the University of Utah's Clinical Genetics Research Program (IRB #7551).

### SNP Genotyping and Quality Control

Forty-nine individual DNA samples were genotyped using Affymetrix 6.0 SNP Array technology (>900,000 SNPs) at Capital Bio Corporation (Beijing, China). We used default parameters for the Birdseed algorithm (version 2) to determine genotypes for all samples (Affymetrix, Santa Clara, CA, USA) and the ERSA program to detect cryptic relatedness between subjects [49]. When a pair of individuals was estimated to be more closely related than first cousin, one member of the pair was excluded from the analyses. Based on these criteria, seven individuals were removed, leaving a total of 42 individuals for subsequent analysis. The genotype data were then combined with data from other Northern Eurasian individuals who were previously genotyped using the same platform [10], [20], [50]. The final dataset contains ∼860,000 SNPs that were genotyped in 369 individuals from 10 Northern Eurasian populations. SNP genotypes of the final dataset are available on our website (http://jorde-lab.genetics.utah.edu/) under Published Data.

### Population Genetic Analysis

Between-population *F_ST_* estimates, neighbor-joining tree construction, pairwise allele-sharing genetic distance calculation, and principal components analysis (PCA) were performed using MATLAB (ver. r2011b) as previously described [50], [51]. Genome-wide admixture estimates were obtained with the *ADMIXTURE* algorithm (version 1.02) [52]. To eliminate the effects of SNPs that are in linkage disequilibrium, we first filtered out SNPs that had pairwise r^2^>0.2 within 50 SNP windows using PLINK [53] as recommended by the authors of *ADMIXTURE*. The pruned data set contains 142,888 SNPs.

### Selection Analyses

The XP-EHH (cross-population extended haplotype homozygosity) [19], iHS (integrated haplotype score) [54], and PBS (population branch statistic) selection scans were performed as previously described [20], [21]. For XP-EHH and PBS selection scans, our test statistic was the maximum score in each 200 kb region [19]. For XPEHH, we used the HapMap CHB and JPT populations as reference populations and calculated XP-EHH at each site using default settings (http://hgdp.uchicago.edu/Software/). For PBS, we used the HapMap CHB and JPT as the first reference population and HapMap CEU as the second. We determined statistical significance for each region from the empirical distribution of the test statistic and selected regions that are significant at the 0.02 level as our candidates. To identify selected loci at intermediate frequencies, we employed the iHS statistic. We summed the integrated EHH in both directions from each SNP until EHH was less than or equal to 0.10 and calculated the iHS score as the log ratio of iHH single-site scores standardized by the population-derived allele frequency. After excluding regions with <5 SNPs, we determined the iHS test statistic for each 200 kb region as the fraction of SNPs in which the absolute value of iHS was >2.0. The expected number of overlapping regions in Mongolian and Tibetan populations was determined using simulation assuming independence of the regions, and the significance level of the observation was determined using the empirical distribution from the simulation.

The multiple regression (MR) analysis was performed as described in Alkorta-Aranburu et al. [55] to identify unusually divergent SNP loci in the DU Mongolian population. Specifically, the observed allele frequencies at all genotyped SNPs in six populations (excluding the Buryat Mongolians and two Tibetan groups) were used to predict the allele frequencies at those loci in DU Mongolians as a linear combination of frequencies in the six populations. The MR Score of a SNP is defined as the Studentized (scaled) residual of the observed vs. predicted allele frequency for the SNP. For each gene, we computed an MR Factor as the proportion of SNPs within its transcribed region plus 10 kb on either side that are in the tail of the MR Score distribution divided by the proportion of tail SNPs for all such gene regions. As in Alkorta-Aranburu et al. [55], we considered three tails of the MR Score distribution (5%, 1% and 0.5%) and computed MR Factors for each level. The MR Factors and empirical p-values (transformed rank statistics) for the top 2% of gene regions with at least 10 genotyped SNPs are shown in Table S4.

To control for potential batch effect in genotype calling, we combined our samples and HapMap samples and performed genotype calling on whole dataset. SNPs that showed less than 50% concordance rate in any given population between the original genotypes and the recalled genotypes were excluded from the PBS and MR analyses.

### Phenotype Collection and Genotype-Phenotype Association Test

[Hb] and hematocrit were determined from venous blood samples using the Mindray Hematology Analyzer (BC-2300, Shenzhen, China). Of the 42 unrelated samples, we were able to obtain [Hb] from 30 non-smoking, healthy individuals. The [Hb] values of 326 Tibetan and 335 Han Chinese female individuals (between ages 16 and 60) collected at a comparable altitude (2664 m) were obtained from a previous study [32].

To determine if [Hb] in DU Mongolians is associated with haplotypes that have undergone selection in Tibetans, we first defined the selected Tibetan haplotypes using three core SNPs in each region as previously described [20] and estimated the copy number of each haplotype in each DU Mongolian individual. We then used a stepwise linear regression model (MATLAB ver. r2011b) to determine the association between [Hb] and the inferred Tibetan haplotype copies of each genic region in DU Mongolians.

### Whole-Genome Sequencing

Whole-genome sequencing was performed by Complete Genomics, Inc. (CGI). The whole-genome sequencing yielded ∼1.6×10^11^ bp of total sequence, with an average coverage of 51.7× for the entire genome. The raw sequences were mapped to the NCBI reference human genome build 37 (hg19), and variant calling was performed by CGI using their variant-calling pipeline (Software version 1.11.0.18). Overall, both alleles were determined for 96.2% of the genome, one of the two alleles for 0.7% of the genome, and neither allele for 3.2% of the genome. The whole-genome variant calls of individuals in the CGI54 panel were obtained from the CGI website (ftp://ftp2.completegenomics.com/). This panel contains 54 unrelated individuals, including all members of the CGI diversity panel (46 individuals), parents in the YRI and the PUR trios (four individuals), and four grandparents in CEU Pedigree_1463. The 1000 Genomes phase 1 variant calls were downloaded from the 1000 Genomes website (ftp://ftp.1000genomes.ebi.ac.uk/vol1/ftp/phase1/).

To validate the mtDNA variants in the Tianjiao1 genome, we performed Sanger sequencing on the HVS1 region and resequenced the entire mitochondrial genome using the Ion Torrent platform. All methods produced identical variant calls and confirmed the whole-genome sequencing results.

The functional impact of the genomic variants was assessed using the Variant Annotation Tool (VAT) in the Variant Annotation, Analysis and Search Tool (VAAST) package [56]. Tianjiao1 variants that are present in the CGI54 panel individuals were excluded using the Variant Selection Tool (VST) in the VAAST package, and variants that overlap the 1000 Genomes phase 1 variants were excluded using tabix (http://samtools.sourceforge.net) [57]. The functional significance of coding SNVs and indels was assessed using SIFT [46] (SIFT Human Coding SNPs: http://sift.jcvi.org/www/SIFT_chr_coords_submit.html; SIFT Human Coding indels: http://sift.jcvi.org/www/SIFT_chr_coords_indels_submit.html).

## Supporting Information

Figure S1 Number of variants in Tianjiao1 and CGI54 panel individuals. Individuals in the diversity panel were grouped by populations. The mean number of variants and standard deviation for each population is shown. Population code: DUM: Tianjiao1; CHB: Han Chinese; JPT: Japanese; GIH: Gujarati; PUR: Puerto Rican; MXL: Mexican-American; TSI: Tuscan; CEU: Utah residents (CEPH) with Northern and Western European ancestry; MKK: Maasai; ASW: African-American; YRI: Yoruba; LWK: Luhya.
(DOCX)

Table S1 Overlap of DU and Buryat Mongolian selection candidate regions at p<0.02 level in iHS and/or XP-EHH selection scans.
(DOCX)

Table S2 Overlap of Tibetan and Mongolian selection candidate regions at p<0.02 level in iHS and/or XP-EHH selection scans.
(DOCX)

Table S3 Top 2% of PBS selection candidate regions identified in DU Mongolians.
(DOCX)

Table S4 Top 2% of selection candidate genes identified in the MR analysis in DU Mongolians.
(DOCX)

Table S5 Stepwise linear regression analysis of [Hb] and Tibetan high-altitude selection candidate gene haplotypes in 26 female DU Mongolians.
(DOCX)

Table S6 Key variants for Tianjiao1 mtDNA and Y-chromosome haplogroup assignments.
(DOCX)

Table S7 SIFT prediction for the effect of Tianjiao1-specific SNVs.
(DOCX)

Table S8 SIFT prediction for the effect of Tianjiao1-specific Indels.
(DOCX)
