# Supplementary figures and images for: Genomic Analysis of Natural Selection and Phenotypic Variation in High-Altitude Mongolians
Source: PLoS Genet. 2013 Jul 18;9(7):e1003634. doi: 10.1371/journal.pgen.1003634 (PMC3715426; doi:10.1371/journal.pgen.1003634)

**Figure S1**

**
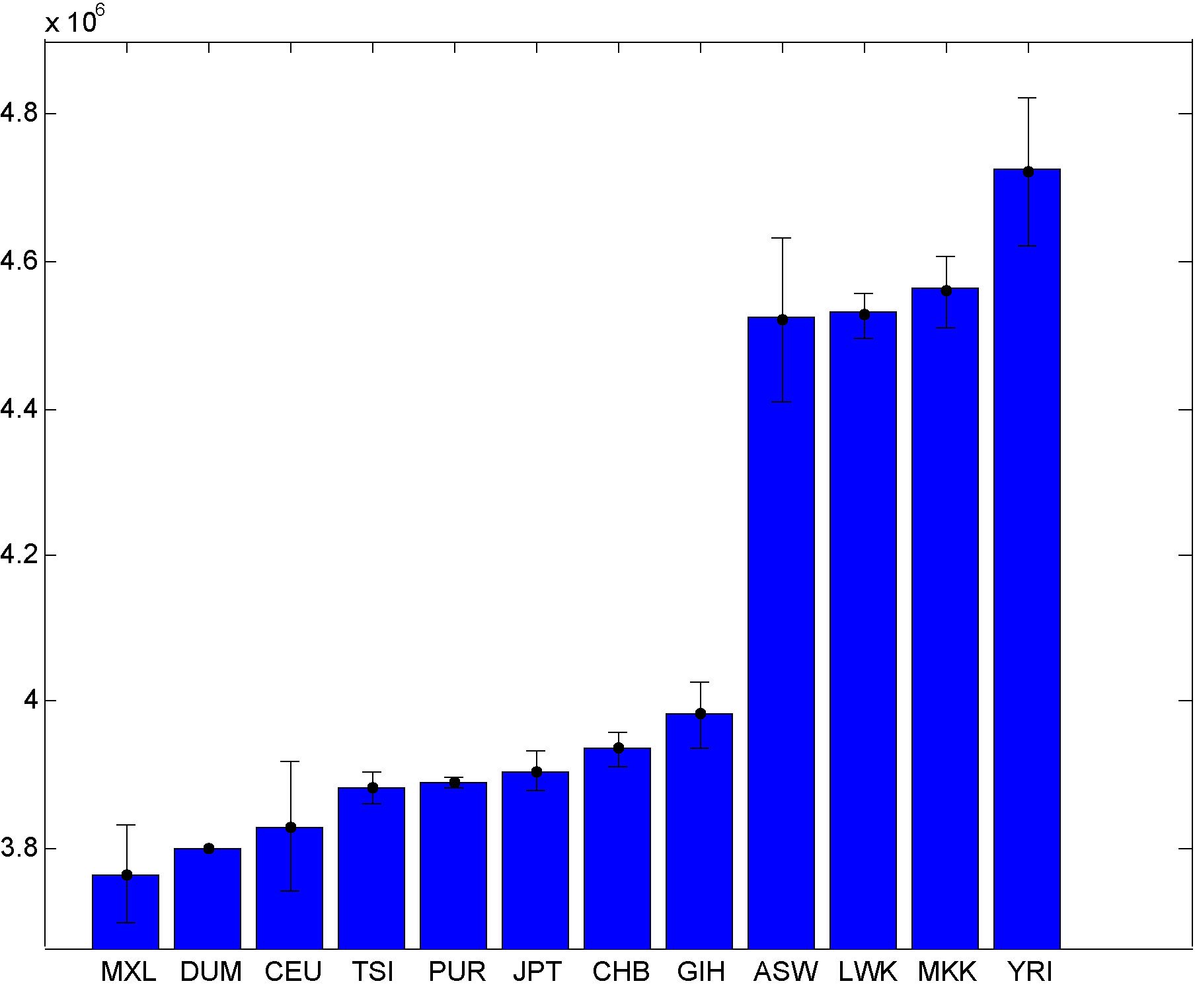
**

Supplement: Figure S1 — Number of variants in Tianjiao1 and CGI54 panel individuals. Individuals in the diversity panel were grouped by populations. The mean number of variants and standard deviation for each population is shown. Population code: DUM: Tianjiao1; CHB: Han Chinese; JPT: Japanese; GIH: Gujarati; PUR: Puerto Rican; MXL: Mexican-American; TSI: Tuscan; CEU: Utah residents (CEPH) with Northern and Western European ancestry; MKK: Maasai; ASW: African-American; YRI: Yoruba; LWK: Luhya. (DOCX) [file pgen.1003634.s001.docx]
