# Supplementary material for: Genomic Analysis of Natural Selection and Phenotypic Variation in High-Altitude Mongolians
Source: PLoS Genet. 2013 Jul 18;9(7):e1003634. doi: 10.1371/journal.pgen.1003634 (PMC3715426; doi:10.1371/journal.pgen.1003634)
Supplement: Table S1 — Overlap of DU and Buryat Mongolian selection candidate regions at p<0.02 level in iHS and/or XP-EHH selection scans. (DOCX) [file pgen.1003634.s002.docx]

**Table S1. Overlap of DU and Buryat Mongolian selection candidate regions at p < 0.02 level in iHS and/or XP-EHH selection scans**

| **Chr** | **200KB Region** | **Gene List** | **DU Mongolian** | **p** | **Buryat Mongolian** | **p** |
| --- | --- | --- | --- | --- | --- | --- |
| chr1 | 377 | SLC44A5 | iHS | 0.0008 | iHS | 0.0148 |
| chr1 | 403 | No Gene | XP-EHH | 0.0012 | XP-EHH | 0.0062 |
| chr1 | 569 | MAGI3 | iHS | 0.0145 | iHS | 0.0003 |
| chr1 | 596 | WARS2,TBX15 | iHS | 0.0112 | iHS | 0.0009 |
| chr1 | 894 | XPR1 | iHS | 0.012 | iHS | 0.0167 |
| chr2 | 58 | GREB1,NTSR2 | XP-EHH | 0.0072 | XP-EHH | 0.0093 |
| chr2 | 416 | No Gene | iHS | 0.0025 | iHS | 0.0011 |
| chr2 | 429 | ST3GAL5,ATOH8 | iHS | - | iHS | 0.0146 |
|  |  |  | XP-EHH | 0.019 | XP-EHH | 0.0052 |
| chr2 | 539 | SLC5A7,LOC729121,RGPD4 | iHS | 0.0196 | iHS | 0.001 |
| chr2 | 541 | SULT1C2,SULT1C3,SULT1C4 | iHS | 0.0013 | iHS | 0.0002 |
| chr2 | 589 | No Gene | XP-EHH | 0.0051 | XP-EHH | 0.013 |
| chr2 | 687 | THSD7B | XP-EHH | 0.0109 | iHS | 0.0108 |
| chr2 | 769 | No Gene | iHS | 0.015 | iHS | 0.0026 |
| chr2 | 891 | PDE11A | iHS | - | iHS | 0.0096 |
|  |  |  | XP-EHH | 0.0149 | XP-EHH | 0.0027 |
| chr2 | 893 | PDE11A,OSBPL6,RBM45 | iHS | 0.0049 | iHS | 0.0018 |
|  |  |  | XP-EHH | - | XP-EHH | 0.0197 |
| chr2 | 948 | COL5A2 | iHS | 0.0027 | XP-EHH | 0.0085 |
| chr2 | 950 | WDR75,SLC40A1 | iHS | 0.0187 | iHS | 0.0032 |
| chr2 | 1020 | RAPH1,ABI2 | iHS | 0.0134 | iHS | 0.0195 |
| chr2 | 1142 | WDR69,SPHKAP | XP-EHH | 0.0192 | XP-EHH | 0.018 |
| chr3 | 327 | MAGI1 | iHS | 0.0159 | iHS | 0.0036 |
| chr3 | 564 | PHLDB2,PLCXD2,CD96 | iHS | 0.0123 | iHS | 0.004 |
| chr3 | 828 | No Gene | iHS | 0.0108 | iHS | 0.0111 |
| chr3 | 829 | No Gene | iHS | 0.003 | iHS | 0.0033 |
| chr3 | 873 | NLGN1 | iHS | 0.0097 | iHS | 0.0067 |
| chr4 | 82 | LDB2 | iHS | 0.0163 | iHS | 0.0169 |
| chr4 | 130 | TBC1D19,RBPJ,CCKAR | XP-EHH | 0.0032 | iHS | 0.0163 |
| chr4 | 170 | No Gene | iHS | 0.0058 | iHS | 0.0075 |
| chr4 | 304 | No Gene | iHS | 0.0069 | iHS | 0.0111 |
| chr4 | 481 | UNC5C,BMPR1B | XP-EHH | 0.0196 | XP-EHH | 0.0047 |
| chr4 | 614 | TMEM155,CCNA2,EXOSC9,ANXA5,LOC100192379,BBS7 | XP-EHH | 0.0061 | iHS | 0.0083 |
| chr4 | 719 | INPP4B | iHS | 0.005 | iHS | 0.0027 |
| chr4 | 720 | No Gene | iHS | 0.0101 | iHS | 0.0173 |
| chr4 | 722 | GAB1 | iHS | 0.0007 | iHS | 0.0022 |
| chr4 | 793 | No Gene | iHS | 0.0018 | iHS | 0.0005 |
| chr4 | 803 | No Gene | iHS | 0.0083 | iHS | 0.0109 |
| chr4 | 846 | DDX60,ANXA10 | iHS | 0.004 | XP-EHH | 0.0163 |
| chr5 | 544 | No Gene | iHS | 0.0114 | iHS | 0.0179 |
| chr5 | 562 | MCC | iHS | 0.0042 | iHS | 0.0046 |
| chr5 | 674 | NEUROG1,CXCL14,C5orf20,TIFAB | XP-EHH | 0.0018 | XP-EHH | 0.0186 |
|  |  |  | iHS | 0.0198 | iHS | 0.0165 |
| chr5 | 678 | TRPC7 | XP-EHH | 0.0189 | XP-EHH | 0.0176 |
| chr5 | 739 | FBXO38,HTR4 | XP-EHH | 0.0139 | XP-EHH | 0.0078 |
| chr6 | 48 | No Gene | iHS | 0.0185 | iHS | 0.0131 |
| chr6 | 133 | ABT1,BTN1A1,HMGN4,ZNF322A | iHS | 0.0051 | iHS | 0.011 |
| chr6 | 174 | ANKS1A,UHRF1BP1,SNRPC,TAF11 | XP-EHH | 0.0156 | XP-EHH | 0.0028 |
| chr6 | 239 | OPN5,C6orf138 | iHS | 0.0108 | iHS | 0.0007 |
| chr6 | 313 | KHDRBS2 | iHS | 0.0182 | iHS | 0.0093 |
| chr6 | 350 | BAI3 | iHS | 0.007 | iHS | 0.0057 |
| chr6 | 417 | No Gene | iHS | 0.0035 | iHS | 0.0071 |
| chr6 | 629 | No Gene | iHS | 0.0038 | iHS | 0.0051 |
| chr7 | 40 | GLCCI1,ICA1 | XP-EHH | 0.0007 | XP-EHH | 0.0079 |
| chr7 | 71 | DGKB | XP-EHH | 0.0045 | XP-EHH | 0.0067 |
| chr7 | 393 | MAGI2 | XP-EHH | 0.0159 | XP-EHH | 0.016 |
| chr7 | 400 | CD36 | XP-EHH | 0.0025 | XP-EHH | 0.0007 |
| chr7 | 493 | MYH16,ARPC1A | iHS | 0.0112 | iHS | - |
|  |  |  | XP-EHH | 0.0132 | XP-EHH | 0.0102 |
| chr7 | 675 | FAM180A,SLC13A4,PL-5283 | XP-EHH | 0.0038 | XP-EHH | 0.0008 |
| chr8 | 22 | CSMD1 | XP-EHH | 0.0036 | XP-EHH | 0.0189 |
| chr8 | 658 | No Gene | XP-EHH | 0.0157 | XP-EHH | 0.001 |
| chr10 | 20 | No Gene | XP-EHH | 0.007 | iHS | 0.0194 |
| chr10 | 286 | No Gene | iHS | 0.0141 | iHS | 0.0012 |
|  |  |  | XP-EHH | - | XP-EHH | 0.0153 |
| chr10 | 295 | No Gene | iHS | 0.0174 | iHS | 0.0147 |
| chr10 | 376 | KIAA0913,NDST2,FUT11,SEC24C,CHCHD1,PLAU,C10orf55,CAMK2G | iHS | 0.0147 | iHS | 0.0178 |
| chr10 | 473 | EXOC6 | iHS | 0.0122 | iHS | 0.0184 |
| chr10 | 474 | EXOC6,CYP26A1,CYP26C1 | iHS | 0.0062 | iHS | 0.0019 |
| chr10 | 475 | MYOF | iHS | 0.0031 | iHS | 0.0076 |
| chr10 | 563 | NCRNA00081,PDCD4,SHOC2 | XP-EHH | 0.0046 | XP-EHH | 0.0002 |
| chr10 | 564 | ADRA2A | XP-EHH | 0.0136 | XP-EHH | 0.0001 |
| chr10 | 609 | No Gene | iHS | - | iHS | 0.0197 |
|  |  |  | XP-EHH | 0.0126 | XP-EHH | 0.0026 |
| chr11 | 199 | No Gene | iHS | 0.0052 | iHS | 0.007 |
| chr11 | 221 | ALX4,EXT2 | XP-EHH | 0.012 | XP-EHH | 0.019 |
| chr11 | 340 | SAPS3 | iHS | 0.0024 | iHS | 0.0028 |
| chr12 | 53 | STYK1,MAGOHB,KLRA1,CSDA | XP-EHH | 0.0194 | XP-EHH | 0.0109 |
| chr12 | 163 | FGD4,YARS2,DNM1L | iHS | 0.0189 | iHS | 0.0162 |
| chr12 | 225 | SLC38A2 | XP-EHH | 0.002 | XP-EHH | 0.0012 |
| chr12 | 226 | No Gene | XP-EHH | 0.009 | XP-EHH | 0.0091 |
| chr13 | 152 | C13orf26 | XP-EHH | 0.0013 | XP-EHH | 0.0128 |
| chr13 | 302 | No Gene | iHS | 0.013 | iHS | 0.0155 |
| chr13 | 483 | MBNL2 | XP-EHH | 0.0095 | XP-EHH | 0.0002 |
| chr13 | 519 | No Gene | iHS | 0.0078 | iHS | 0.013 |
| chr14 | 313 | KCNH5,RHOJ | iHS | - | iHS | 0.0131 |
|  |  |  | XP-EHH | 0.0014 | XP-EHH | 0.0022 |
| chr14 | 343 | WDR22,EXDL2,GALNTL1 | iHS | 0.0004 | iHS | 0.0013 |
| chr14 | 434 | No Gene | iHS | 0.0118 | iHS | 0.0192 |
| chr15 | 135 | KIAA0574,APBA2 | XP-EHH | 0.0005 | XP-EHH | 0.0145 |
| chr15 | 160 | AVEN,C15orf24,CHRM5,PGBD4 | XP-EHH | 0.0011 | XP-EHH | 0.0081 |
| chr15 | 206 | TGM5,TGM7,CCNDBP1,EPB42,TMEM62 | iHS | 0.0067 | iHS | 0.012 |
| chr15 | 217 | C15orf21,SPATA5L1,SLC30A4,GATM,C15orf48 | iHS | 0.0163 | iHS | 0.0072 |
| chr15 | 329 | LBXCOR1,MAP2K5 | iHS | 0.0117 | iHS | 0.006 |
| chr15 | 432 | NTRK3 | iHS | 0.0193 | iHS | 0.0144 |
| chr16 | 149 | DOC2A,PPP4C,ASPHD1,TMEM219,FAM57B,FLJ25404,TAOK2,KCTD13,SEZ6L2,ALDOA,HIRIP3,INO80E | iHS | 0.0034 | iHS | 0.0042 |
| chr16 | 321 | No Gene | iHS | 0.0002 | iHS | 0.0052 |
| chr16 | 403 | MPHOSPH6,HSD17B2,SDR42E1 | XP-EHH | 0.0141 | XP-EHH | 0.017 |
| chr20 | 0 | C20orf96,DEFB127,DEFB128,DEFB132,DEFB129,DEFB125,DEFB126 | XP-EHH | 0.0062 | XP-EHH | 0.0018 |
| chr20 | 167 | GDF5,UQCC,CEP250,ERGIC3 | iHS | 0.0021 | iHS | 0.0113 |
| chr20 | 293 | No Gene | XP-EHH | 0.0009 | XP-EHH | 0.0121 |
| chr21 | 79 | No Gene | XP-EHH | 0.0112 | iHS | 0.0052 |
| chr21 | 208 | FAM3B,TMPRSS2,MX1,MX2 | XP-EHH | 0.0006 | XP-EHH | 0.0059 |
| chr22 | 242 | BRD1 | iHS | - | iHS | 0.0053 |
|  |  |  | XP-EHH | 0.0031 | XP-EHH | 0.0015 |
