# Supplementary material for: Genomic Analysis of Natural Selection and Phenotypic Variation in High-Altitude Mongolians
Source: PLoS Genet. 2013 Jul 18;9(7):e1003634. doi: 10.1371/journal.pgen.1003634 (PMC3715426; doi:10.1371/journal.pgen.1003634)
Supplement: Table S2 — Overlap of Tibetan and Mongolian selection candidate regions at p<0.02 level in iHS and/or XP-EHH selection scans. (DOCX) [file pgen.1003634.s003.docx]

**Table S2. Overlap of Tibetan and Mongolian selection candidate regions at p < 0.02 level in iHS and/or XP-EHH selection scans**

| **Chr** | **200KB Region** | **Gene List** | **DU Mongolian** | **p** | **Buryat Mongolian** | **p** | **TTR Tibetan** | **p** | **Maduo Tibetan** | **p** |
| --- | --- | --- | --- | --- | --- | --- | --- | --- | --- | --- |
| chr1 | 117 | TCEA3,ZNF436,HNRNPR,C1orf213 | iHS | 0.0079 |  |  |  |  | iHS | 0.0141 |
| chr1 | 251 | AGBL4,ELAVL4 |  |  | iHS | 0.0032 | iHS | 0.0139 | iHS | 0.0083 |
|  |  |  |  |  |  |  | XP-EHH | 0.0124 |  |  |
| chr1 | 252 | ELAVL4 | iHS | 0.0143 |  |  | XP-EHH | 0.0136 |  |  |
| chr1 | 254 | FAF1 | iHS | 0.0028 |  |  | XP-EHH | 0.0165 |  |  |
| chr1 | 377 | SLC44A5 | iHS | 0.0008 | iHS | 0.0148 | XP-EHH | 0.0081 |  |  |
| chr1 | 411 | LPHN2 |  |  | iHS | 0.0117 | iHS | 0.0128 |  |  |
| chr1 | 569 | MAGI3 | iHS | 0.0145 | iHS | 0.0003 | iHS | 0.0124 |  |  |
| chr1 | 767 | GBAP,PKLR^b^,C1orf104,SCAMP3,MTX1,GBA,HCN3,TRIM46,KRTCAP2,FDPS,MUC1,C1orf2,CLK2,THBS3,ASH1L,RUSC1 | iHS | 0.0092 |  |  | iHS | 0.0043 | iHS | 0.02 |
| chr1 | 799 | DUSP12,FCGR3B,FCRLA,LOC641311,FCGR2B,FCRLB,HSPA7,FCGR2C | iHS | 0.0093 |  |  | iHS | 0.0068 |  |  |
| chr1 | 890 | FAM163A,TOR1AIP2,IFRG15,CEP350,TOR1AIP1 | iHS | 0.0095 |  |  | iHS | 0.0142 | iHS | 0.0035 |
| chr1 | 894 | XPR1 | iHS | 0.012 | iHS | 0.0167 |  |  | iHS | 0.0135 |
| chr1 | 898 | CACNA1E | XP-EHH | 0.0039 |  |  |  |  | iHS | 0.0095 |
| chr1 | 1026 | PFKFB2,C4BPB,C4BPA,FCAMR,YOD1,C1orf116 |  |  | iHS | 0.0073 |  |  | iHS | 0.0115 |
| chr1 | 1070 | USH2A | XP-EHH | 0.0089 |  |  |  |  | XP-EHH | 0.0131 |
| chr1 | 1124 | ITPKB,C1orf95 |  |  | XP-EHH | 0.0039 |  |  | XP-EHH | 0.0064 |
| chr2 | 88 | VSNL1,SMC6,GEN1 | iHS | 0.0013 |  |  | XP-EHH | 0.0129 | iHS | 0.0023 |
| chr2 | 162 | BIRC6 | XP-EHH | 0.0117 |  |  |  |  | XP-EHH | 0.0122 |
| chr2 | 163 | BIRC6,TTC27 | XP-EHH | 0.0066 |  |  |  |  | XP-EHH | 0.0062 |
| chr2 | 231 | PRKCE,EPAS1^b^ | XP-EHH | 0.0069 |  |  |  |  | XP-EHH | 0.0015 |
| chr2 | 236 | C2orf61,CALM2 |  |  | XP-EHH | 0.016 |  |  | XP-EHH | 0.0079 |
| chr2 | 342 | PLEK,APLF,CNRIP1,FBXO48 | iHS | 0.0057 |  |  | iHS | 0.0145 |  |  |
| chr2 | 430 | PTCD3,POLR1A |  |  | iHS | 0.0081 | XP-EHH | 0.0027 |  |  |
|  |  |  |  |  | XP-EHH | 0.01 |  |  |  |  |
| chr2 | 522 | LOC150568 | XP-EHH | 0.0015 |  |  |  |  | XP-EHH | 0.0066 |
| chr2 | 541 | SULT1C3,SULT1C2,SULT1C4 | iHS | 0.0013 | iHS | 0.0002 |  |  | iHS | 0.0105 |
| chr2 | 544 | EDAR,CCDC138 | iHS | 0.0035 |  |  | iHS | 0.0164 |  |  |
| chr2 | 772 | GALNT13 | iHS | 0.01 |  |  |  |  | iHS | 0.0073 |
| chr2 | 793 | CCDC148,UPP2 |  |  | iHS | 0.0128 | iHS | 0.0172 |  |  |
| chr2 | 838 | XIRP2 | XP-EHH | 0.0134 |  |  |  |  | XP-EHH | 0.0087 |
| chr2 | 891 | PDE11A | XP-EHH | 0.0149 | iHS | 0.0096 | XP-EHH | 0.0101 |  |  |
|  |  |  |  |  | XP-EHH | 0.0027 |  |  |  |  |
| chr2 | 916 | DNAJC10 | XP-EHH | 0.0003 |  |  |  |  | XP-EHH | 0.0022 |
| chr2 | 951 | PMS1,ORMDL1,ANKAR,OSGEPL1,ASNSD1 |  |  | iHS | 0.0118 | XP-EHH | 0.0036 | iHS | 0.0118 |
| chr2 | 1020 | RAPH1,ABI2 | iHS | 0.0134 | iHS | 0.0195 | iHS | 0.0117 |  |  |
| chr2 | 1094 | C2orf62,GPBAR1,SLC11A1,TMBIM1,PNKD,CTDSP1,AAMP,ARPC2,VIL1 | iHS | 0.019 |  |  | iHS | 0.0168 | iHS | 0.0056 |
| chr2 | 1095 | USP37,RQCD1,PLCD4,VIL1 |  |  | iHS | 0.0181 | iHS | 0.0099 |  |  |
| chr3 | 62 | PPARG,TSEN2,MKRN2 | iHS | 0.0071 |  |  | iHS | 0.0148 | iHS | 0.0178 |
| chr3 | 127 | RARB |  |  | XP-EHH | 0.0038 | XP-EHH | 0.0006 |  |  |
| chr3 | 242 | PLXNB1,CCDC51,ATRIP,TREX1,PFKFB4,FBXW12,UCN2,CCDC72,SHISA5,COL7A1 | iHS | 0.0053 |  |  |  |  | iHS | 0.0031 |
| chr3 | 327 | MAGI1 | iHS | 0.0159 | iHS | 0.0036 |  |  | iHS | 0.0114 |
| chr3 | 490 | EPHA6 |  |  | iHS | 0.0004 |  |  | iHS | 0.0168 |
| chr3 | 543 | BBX |  |  | iHS | 0.0029 | iHS | 0.0028 |  |  |
| chr3 | 551 | MORC1 |  |  | iHS | 0.0122 |  |  | iHS | 0.0121 |
| chr3 | 552 | DPPA2,DPPA4 | iHS | 0.0024 |  |  |  |  | iHS | 0.0082 |
| chr3 | 579 | ZBTB20 | iHS | 0.0019 |  |  | iHS | 0.0135 | iHS | 0.0037 |
| chr3 | 789 | TIPARP | iHS | 0.0126 |  |  |  |  | iHS | 0.013 |
| chr3 | 793 | VEPH1,PTX3,C3orf55 |  |  | XP-EHH | 0.0069 |  |  | XP-EHH | 0.0107 |
| chr3 | 813 | OTOL1 | iHS | 0.0189 |  |  |  |  | iHS | 0.0126 |
| chr3 | 844 | SERPINI1,WDR49,PDCD10 |  |  | iHS | 0.0159 | iHS | 0.0019 | iHS | 0.0196 |
| chr4 | 99 | SLIT2 | iHS | 0.0166 |  |  |  |  | iHS | 0.0086 |
| chr4 | 107 | NCRNA00099,KCNIP4 | iHS | 0.0152 |  |  | iHS | 0.0027 | iHS | 0.0007 |
| chr4 | 206 | LIMCH1 | iHS | 0.0157 |  |  | iHS | 0.0089 |  |  |
| chr4 | 207 | PHOX2B |  |  | iHS | 0.0185 | iHS | 0.0033 |  |  |
| chr4 | 481 | UNC5C,BMPR1B | XP-EHH | 0.0196 | XP-EHH | 0.0047 | XP-EHH | 0.0111 |  |  |
| **chr4 ^a^** | **614** | **TMEM155,CCNA2,EXOSC9,ANXA5,LOC100192379,BBS7** | **XP-EHH** | **0.0061** | **iHS** | **0.0083** | **iHS** | **0.0179** | **iHS** | **0.0044** |
|  |  |  |  |  |  |  |  |  | **XP-EHH** | **0.004** |
| chr4 | 615 | TRPC3,BBS7 |  |  | iHS | 0.0153 |  |  | iHS | 0.0029 |
|  |  |  |  |  |  |  |  |  | XP-EHH | 0.0027 |
| chr4 | 718 | INPP4B |  |  | iHS | 0.0102 | iHS | 0.0105 | iHS | 0.0175 |
| **chr4** | **719** | **INPP4B** | **iHS** | **0.005** | **iHS** | **0.0027** | **iHS** | **0.0051** | **iHS** | **0.0053** |
| chr4 | 721 | USP38 | iHS | 0.0005 |  |  | iHS | 0.0053 | iHS | 0.0025 |
| chr4 | 722 | GAB1 | iHS | 0.0007 | iHS | 0.0022 | iHS | 0.0192 |  |  |
| chr4 | 743 | EDNRA^c^,TMEM184C,LOC90826 |  |  | iHS | 0.0121 |  |  | iHS | 0.0118 |
|  |  |  |  |  |  |  |  |  | XP-EHH | 0.0078 |
| chr4 | 792 | GRIA2 | iHS | 0.0016 |  |  | iHS | 0.0152 |  |  |
| chr4 | 802 | RAPGEF2 | iHS | 0.002 |  |  | iHS | 0.0055 | iHS | 0.0005 |
| chr5 | 135 | CDH9 | iHS | 0.0192 |  |  |  |  | iHS | 0.0197 |
| chr5 | 216 | ZNF131,HMGCS1,MGC42105 |  |  | iHS | 0.018 |  |  | iHS | 0.0156 |
| chr5 | 323 | ADAMTS6 | iHS | 0.0063 |  |  |  |  | iHS | 0.0155 |
| chr5 | 501 | ST8SIA4 | XP-EHH | 0.0129 |  |  | iHS | 0.0067 |  |  |
| **chr5** | **562** | **MCC** | **iHS** | **0.0042** | **iHS** | **0.0046** | **iHS** | **0.0017** | **iHS** | **0.0027** |
|  |  |  |  |  |  |  | **XP-EHH** | **0.0063** | **XP-EHH** | **0.0164** |
| chr6 | 133 | ABT1,BTN1A1,HMGN4,ZNF322A | iHS | 0.0051 | iHS | 0.011 | iHS | 0.0046 |  |  |
| chr6 | 157 | HCP5,MICA,HLA-B,MICB,HCG26 |  |  | iHS | 0.0095 | iHS | 0.0069 | iHS | 0.0074 |
| chr6 | 162 | BTNL2,HLA-DRA,C6orf10,HLA-DRB5 | iHS | 0.0123 |  |  | iHS | 0.0098 |  |  |
| chr6 | 238 | CD2AP,GPR111,GPR115 |  |  | iHS | 0.0067 |  |  | iHS | 0.001 |
|  |  |  |  |  |  |  |  |  |  |  |
| chr6 | 239 | OPN5,C6orf138 | iHS | 0.0108 | iHS | 0.0007 |  |  | iHS | 0.0152 |
| chr6 | 313 | KHDRBS2 | iHS | 0.0182 | iHS | 0.0093 | iHS | 0.0136 |  |  |
| chr6 | 400 | HMGN3 | iHS | 0.0175 |  |  |  |  | iHS | 0.0199 |
| chr6 | 418 | UBE2CBP | iHS | 0.0115 |  |  | iHS | 0.0073 |  |  |
| chr6 | 544 | LACE1,FOXO3 | iHS | 0.0075 |  |  | iHS | 0.0037 |  |  |
| chr6 | 596 | ASF1A,FAM184A,MCM9 | iHS | 0.0109 |  |  | iHS | 0.0098 | iHS | 0.003 |
| chr6 | 724 | UTRN |  |  | XP-EHH | 0.0137 |  |  | XP-EHH | 0.0132 |
| chr6 | 725 | UTRN |  |  | XP-EHH | 0.0031 |  |  | XP-EHH | 0.0005 |
| chr7 | 19 | SDK1 |  |  | iHS | 0.0048 | XP-EHH | 0.0119 |  |  |
| chr7 | 101 | MACC1,ITGB8 | XP-EHH | 0.0172 |  |  |  |  | XP-EHH | 0.0042 |
| chr7 | 189 | EPDR1,TXNDC3,SFRP4 | XP-EHH | 0.0002 |  |  |  |  | XP-EHH | 0.0069 |
| chr7 | 325 | GUSB,VKORC1L1,ASL | iHS | 0.0071 |  |  |  |  | iHS | 0.0057 |
| chr7 | 412 | PCLO |  |  | iHS | 0.009 |  |  | XP-EHH | 0.0032 |
| chr7 | 562 | LOC401397,GPR85 | iHS | 0.0009 |  |  |  |  | iHS | 0.0059 |
| chr7 | 645 | NRF1 |  |  | iHS | 0.0077 |  |  | iHS | 0.0164 |
| chr7 | 646 | UBE2H |  |  | iHS | 0.0134 | iHS | 0.0139 |  |  |
| chr7 | 664 | EXOC4 | iHS | 0.0115 |  |  |  |  | iHS | 0.0139 |
| chr7 | 711 | TRPV6,C7orf34,EPHB6,KEL,TRPV5 | iHS | 0.0057 |  |  | iHS | 0.0036 | iHS | 0.0054 |
| chr9 | 139 | LINGO2 | XP-EHH | 0.0071 |  |  |  |  | iHS | 0.0171 |
| chr9 | 359 | MAMDC2 | XP-EHH | 0.0034 |  |  | iHS | 0.019 | XP-EHH | 0.0025 |
|  |  |  | iHS | 0.0148 |  |  |  |  | iHS | 0.0116 |
| chr9 | 451 | NXNL2,SPIN1 |  |  | iHS | 0.02 | iHS | 0.0133 | iHS | 0.0167 |
| chr9 | 478 | BARX1 | XP-EHH | 0.0033 |  |  |  |  | XP-EHH | 0.0072 |
| chr9 | 479 | PTPDC1 | XP-EHH | 0.0042 |  |  |  |  | XP-EHH | 0.0053 |
| chr9 | 627 | DENND1A | iHS | 0.0033 |  |  | iHS | 0.0009 |  |  |
| chr9 | 649 | CIZ1,NAIF1,LCN2,C9orf16,LOC389791,SLC25A25,PTGES2 | iHS | 0.0088 |  |  | iHS | 0.0158 |  |  |
| chr10 | 19 | KLF6 | XP-EHH | 0.0179 |  |  |  |  | XP-EHH | 0.0028 |
| chr10 | 84 | RSU1,CUBN |  |  | XP-EHH | 0.011 |  |  | XP-EHH | 0.0125 |
| chr10 | 128 | GPR158 | iHS | 0.02 |  |  | XP-EHH | 0.0118 |  |  |
| chr10 | 271 | MBL2 |  |  | XP-EHH | 0.0024 |  |  | XP-EHH | 0.0198 |
| chr10 | 276 | PCDH15 |  |  | iHS | 0.0125 | iHS | 0.0176 |  |  |
| chr10 | 397 | RPS24,POLR3A | XP-EHH | 0.0149 |  |  |  |  | XP-EHH | 0.0148 |
| chr10 | 454 | LIPA,CH25H | iHS | 0.009 |  |  | iHS | 0.0179 |  |  |
| **chr10** | **475** | **MYOF** | **iHS** | **0.0031** | **iHS** | **0.0076** | **iHS** | **0.011** | **iHS** | **0.0143** |
| chr10 | 563 | NCRNA00081,PDCD4,SHOC2 | XP-EHH | 0.0046 | XP-EHH | 0.0002 | XP-EHH | 0.0035 |  |  |
| chr10 | 675 | C10orf125,PRAP1,CALY,ECHS1,CYP2E1^b^,SPRN,PAOX,MTG1,LOC619207 | iHS | 0.0045 |  |  |  |  | iHS | 0.0148 |
|  |  |  | XP-EHH | 0.0017 |  |  |  |  |  |  |
| chr10 | 676 | FRG2B,SYCE1,DUX4,CYP2E1^b^,LOC728410,LOC653544,LOC653545,LOC653543 | XP-EHH | 0.0026 |  |  |  |  | XP-EHH | 0.0074 |
| chr11 | 218 | HSD17B12 | iHS | 0.0096 |  |  | iHS | 0.0104 |  |  |
| chr11 | 221 | ALX4,EXT2 | XP-EHH | 0.012 | XP-EHH | 0.019 |  |  | XP-EHH | 0.0178 |
| chr11 | 283 | APLNR,LRRC55 |  |  | iHS | 0.015 |  |  | iHS | 0.0183 |
|  |  |  |  |  |  |  |  |  | XP-EHH | 0.0112 |
| chr11 | 284 | P2RX3,PRG3,SLC43A3,RTN4RL2,SSRP1,TNKS1BP1,PRG2 |  |  | iHS | 0.0127 |  |  | iHS | 0.0101 |
| chr11 | 544 | C11orf87 |  |  | XP-EHH | 0.0154 |  |  | XP-EHH | 0.0102 |
| chr12 | 5 | ERC1 | iHS | 0.0005 |  |  | iHS | 0.0122 |  |  |
|  |  |  |  |  |  |  | XP-EHH | 0.0086 |  |  |
| chr12 | 46 | PZP,LOC642846 | XP-EHH | 0.0091 |  |  | iHS | 0.0131 | iHS | 0.0107 |
| chr12 | 61 | BCL2L14,LOH12CR2,LRP6,MANSC1 | XP-EHH | 0.0059 |  |  | iHS | 0.0047 | iHS | 0.0028 |
|  |  |  |  |  |  |  |  |  | XP-EHH | 0.0118 |
| chr12 | 211 | ADAMTS20 | iHS | 0.0192 |  |  | XP-EHH | 0.0175 |  |  |
|  |  |  | XP-EHH | 0.0119 |  |  |  |  |  |  |
| chr12 | 212 | PUS7L,IRAK4,TWF1,TMEM117 | iHS | 0.0029 |  |  | XP-EHH | 0.0178 |  |  |
|  |  |  | XP-EHH | 0.013 |  |  |  |  |  |  |
| chr12 | 265 | GTSF1,COPZ1,NCKAP1L,ITGA5,ZNF385A,GPR84 | iHS | 0.0047 |  |  | iHS | 0.0198 |  |  |
| chr12 | 470 | FGD6,VEZT | XP-EHH | 0.0092 |  |  |  |  | XP-EHH | 0.0079 |
| chr12 | 489 | ANKS1B | iHS | 0.0153 |  |  |  |  | iHS | 0.0173 |
| chr12 | 594 | CCDC64 | XP-EHH | 0.0029 |  |  |  |  | XP-EHH | 0.0048 |
| chr13 | 330 | PCDH9 | iHS | 0.0065 |  |  | iHS | 0.0172 |  |  |
| chr13 | 426 | SLITRK6 |  |  | iHS | 0.014 |  |  | iHS | 0.0147 |
| chr13 | 504 | NALCN,ITGBL1 | XP-EHH | 0.0102 |  |  |  |  | XP-EHH | 0.0195 |
| chr14 | 344 | ERH,SLC39A9,GALNTL1 | iHS | 0.001 |  |  |  |  | iHS | 0.0151 |
| chr14 | 383 | TMEM63C,KIAA1737,ZDHHC22 | XP-EHH | 0.0133 |  |  |  |  | XP-EHH | 0.012 |
| chr15 | 145 | TRPM1,MTMR10,MTMR15 | iHS | 0.0017 |  |  | iHS | 0.008 |  |  |
| chr15 | 206 | TGM5,TGM7,CCNDBP1,EPB42,TMEM62 | iHS | 0.0067 | iHS | 0.012 | iHS | 0.01 |  |  |
| chr15 | 217 | C15orf21,SPATA5L1,SLC30A4,GATM,C15orf48 | iHS | 0.0163 | iHS | 0.0072 | iHS | 0.0031 |  |  |
|  |  |  | iHS | 0.0163 |  |  | XP-EHH | 0.0037 |  |  |
| chr15 | 309 | DAPK2,HERC1 | iHS | 0.0068 |  |  | iHS | 0.0026 |  |  |
| chr15 | 310 | DAPK2,SNX1,FAM96A | iHS | 0.0049 |  |  | iHS | 0.0013 |  |  |
| chr15 | 311 | CSNK1G1,SNX1,SNX22,PPIB | iHS | 0.0037 |  |  | iHS | 0.004 |  |  |
| **chr15** | **329** | **LBXCOR1,MAP2K5** | **iHS** | **0.0117** | **iHS** | **0.006** | **iHS** | **0.0025** | **iHS** | **0.0018** |
| chr15 | 456 | RGMA,CHD2 | iHS | 0.003 |  |  | iHS | 0.0061 | iHS | 0.0036 |
| chr16 | 8 | HN1L,IGFALS,SPSB3,EME2,NUBP2,MRPS34,NME3,IFT140,HAGH,MAPK8IP3,CRAMP1L |  |  | XP-EHH | 0.0016 |  |  | XP-EHH | 0.0001 |
| chr16 | 23 | ROGDI,C16orf71,ANKS3,NUDT16L1,N-PAC,ZNF500,SEPT12,FAM100A,MGRN1 | iHS | 0.0135 |  |  | iHS | 0.0132 |  |  |
| chr16 | 78 | NDE1,MYH11,KIAA0430 | XP-EHH | 0.0018 |  |  |  |  | XP-EHH | 0.0035 |
| chr16 | 154 | BCL7C,STX1B,NCRNA00095,ZNF646,CTF1,FBXL19,HSD3B7,ORAI3,ZNF668,SETD1A,STX4 | iHS | 0.0156 |  |  | XP-EHH | 0.012 |  |  |
| chr16 | 260 | RBL2,AKTIP,RPGRIP1L |  |  | XP-EHH | 0.0082 | iHS | 0.018 |  |  |
| chr16 | 408 | CDH13 |  |  | XP-EHH | 0.0035 |  |  | XP-EHH | 0.0173 |
| chr16 | 443 | PRDM7,GAS8,DBNDD1,C16orf3 |  |  | iHS | 0.0015 |  |  | iHS | 0.006 |
| chr17 | 99 | AKAP10,SPECC1 | iHS | 0.006 |  |  | iHS | 0.0014 |  |  |
| chr17 | 100 | SPECC1,CCDC144C | iHS | 0.0016 |  |  | iHS | 0.0009 |  |  |
| chr17 | 121 | SEZ6,PIPOX,DHRS13,ERAL1,FLOT2,PHF12 |  |  | iHS | 0.0074 | iHS | 0.0127 |  |  |
| chr20 | 95 | LOC100130264,SLC24A3 |  |  | iHS | 0.0149 | iHS | 0.0181 | iHS | 0.0068 |
| chr20 | 112 | FOXA2 |  |  | iHS | 0.0037 |  |  | iHS | 0.0099 |
| chr20 | 166 | FAM83C,EIF6,UQCC,PROCR,MMP24 | iHS | 0.0082 |  |  | iHS | 0.0111 | iHS | 0.0019 |
| **chr20** | **167** | **UQCC,GDF5,CEP250,ERGIC3** | **iHS** | **0.0021** | **iHS** | **0.0113** | **iHS** | **0.0015** | **iHS** | **0.0022** |
| chr20 | 170 | SCAND1,PHF20,EPB41L1,C20orf152 | iHS | 0.0001 |  |  |  |  | iHS | 0.0024 |
| chr20 | 185 | DHX35,FAM83D |  |  | XP-EHH | 0.0134 |  |  | iHS | 0.0143 |
| chr20 | 240 | TMEM189,TMEM189-UBE2V1,SNAI1,UBE2V1,RNF114 | XP-EHH | 0.0063 |  |  |  |  | XP-EHH | 0.0099 |
| chr21 | 221 | C21orf33,AGPAT3,TRAPPC10,PWP2 |  |  | XP-EHH | 0.02 |  |  | iHS | 0.0172 |
| chr22 | 86 | DGCR6,PRODH,DGCR5,DGCR9 | iHS | 0.014 |  |  | iHS | 0.012 |  |  |
| chr22 | 174 | APOL4,APOL2,APOL3,APOL1 | iHS | 0.0026 |  |  |  |  | iHS | 0.0172 |

^a^ Candidate regions that are identified in all four populations are in bold.

^b^ Highlighted as high-altitude selection candidates in Tibetans (Simonson et al. 2010; Yi et al. 2010; Wuren et al. submitted) and exclusively in DU Mongolians

^c^ Highlighted as high-altitude selection candidates in Tibetans (Simonson et al. 2010) and Buryat Mongolians, but not DU Mongolians
