# Supplementary material for: Genomic Analysis of Natural Selection and Phenotypic Variation in High-Altitude Mongolians
Source: PLoS Genet. 2013 Jul 18;9(7):e1003634. doi: 10.1371/journal.pgen.1003634 (PMC3715426; doi:10.1371/journal.pgen.1003634)
Supplement: Table S3 — Top 2% of PBS selection candidate regions identified in DU Mongolians. (DOCX) [file pgen.1003634.s004.docx]

**Table S3. Top 2% of PBS selection candidate regions identified in DU Mongolians**

| **Chr** | **200 KB region** | **Gene list** | **p** |
| --- | --- | --- | --- |
| chr4 | 345 | UGT2B17,UGT2B15,TMPRSS11E2,TMPRSS11E | 0.0001 |
| chr8 | 453 | No genes in this region | 0.0001 |
| chr1 | 1049 | SLC30A1,NEK2,LPGAT1 | 0.0002 |
| chr9 | 149 | No genes in this region | 0.0003 |
| chr6 | 623 | NKAIN2 | 0.0004 |
| chr2 | 790 | ACVR1C,CYTIP | 0.0004 |
| chr11 | 179 | LDLRAD3 | 0.0005 |
| chr12 | 214 | TMEM117 | 0.0006 |
| chr7 | 247 | No genes in this region | 0.0007 |
| chr12 | 579 | TESC,FBXW8,HRK | 0.0007 |
| chr21 | 82 | C21orf34 | 0.0008 |
| chr12 | 500 | SLC5A8,ANO4,UTP20 | 0.0009 |
| chr1 | 579 | VANGL1 | 0.0010 |
| chr6 | 581 | FRK | 0.0010 |
| chr6 | 224 | SUPT3H | 0.0011 |
| chr2 | 395 | REG1B,REG3G | 0.0012 |
| chr8 | 82 | No genes in this region | 0.0013 |
| chr9 | 374 | ANXA1 | 0.0013 |
| chr5 | 311 | No genes in this region | 0.0014 |
| chr15 | 421 | No genes in this region | 0.0015 |
| chr14 | 139 | No genes in this region | 0.0016 |
| chr17 | 305 | CCDC46 | 0.0016 |
| chr4 | 301 | No genes in this region | 0.0017 |
| chr4 | 735 | ZNF827 | 0.0018 |
| chr7 | 395 | No genes in this region | 0.0019 |
| chr9 | 504 | COL15A1,TGFBR1 | 0.0019 |
| chr18 | 331 | No genes in this region | 0.0020 |
| chr3 | 720 | PCOLCE2,PAQR9,TRPC1 | 0.0021 |
| chr16 | 112 | LOC23117,LOC100132247,LOC100190986,LOC653786 | 0.0022 |
| chr16 | 107 | IGSF6,IMAA,METTL9,OTOA | 0.0022 |
| chr15 | 308 | FBXL22,HERC1,USP3 | 0.0023 |
| chr2 | 525 | MRPS9 | 0.0024 |
| chr11 | 305 | RPLP0P2,C11orf66,SYT7 | 0.0025 |
| chr11 | 354 | NADSYN1,KRTAP5-10,KRTAP5-7,KRTAP5-8,DHCR7,KRTAP5-11,KRTAP5-9 | 0.0025 |
| chr17 | 45 | NTN1,STX8 | 0.0026 |
| chr2 | 587 | No genes in this region | 0.0027 |
| chr2 | 19 | No genes in this region | 0.0028 |
| chr2 | 894 | OSBPL6 | 0.0028 |
| chr15 | 307 | USP3,CA12 | 0.0029 |
| chr3 | 12 | CNTN4 | 0.0030 |
| chr12 | 61 | BCL2L14,LOH12CR2,LRP6,MANSC1 | 0.0031 |
| chr9 | 478 | BARX1 | 0.0031 |
| chr18 | 347 | No genes in this region | 0.0032 |
| chr7 | 268 | No genes in this region | 0.0033 |
| chr10 | 315 | C10orf107 | 0.0034 |
| chr22 | 123 | MYO18B | 0.0034 |
| chr13 | 125 | ATP8A2 | 0.0035 |
| chr6 | 504 | SIM1 | 0.0036 |
| chr2 | 854 | MYO3B | 0.0037 |
| chr4 | 726 | GYPA | 0.0037 |
| chr19 | 101 | ZNF826,LOC284441 | 0.0038 |
| chr2 | 260 | No genes in this region | 0.0039 |
| chr8 | 16 | CSMD1 | 0.0040 |
| chr8 | 509 | YWHAZ,PABPC1 | 0.0040 |
| chr15 | 401 | FAM154B,EFTUD1 | 0.0041 |
| chr2 | 231 | PRKCE,EPAS1 | 0.0042 |
| chr5 | 612 | PRDM6,PPIC | 0.0043 |
| chr3 | 849 | No genes in this region | 0.0043 |
| chr20 | 217 | SYS1,WFDC2,SYS1-DBNDD2,PIGT,DBNDD2,SPINT3,SDC4,TP53TG5,WFDC6 | 0.0044 |
| chr20 | 308 | PRIC285,C20orf195,EEF1A2,RTEL1,C20orf149,SRMS,PTK6,GMEB2,TNFRSF6B,STMN3 | 0.0045 |
| chr5 | 710 | FGF1,ARHGAP26 | 0.0046 |
| chr15 | 254 | ONECUT1 | 0.0046 |
| chr13 | 462 | No genes in this region | 0.0047 |
| chr13 | 461 | GPC5 | 0.0048 |
| chr3 | 61 | PPARG,GSTM1L,SYN2 | 0.0049 |
| chr16 | 119 | PRKCB | 0.0049 |
| chr7 | 36 | COL28A1,C1GALT1 | 0.0050 |
| chr1 | 813 | PBX1 | 0.0051 |
| chr14 | 383 | TMEM63C,KIAA1737,ZDHHC22 | 0.0052 |
| chr6 | 844 | SMOC2 | 0.0052 |
| chr13 | 501 | No genes in this region | 0.0053 |
| chr3 | 571 | GTPBP8,C3orf17 | 0.0054 |
| chr3 | 623 | PTPLB,ADCY5 | 0.0055 |
| chr12 | 569 | No genes in this region | 0.0055 |
| chr13 | 504 | NALCN,ITGBL1 | 0.0056 |
| chr12 | 633 | No genes in this region | 0.0057 |
| chr12 | 68 | GRIN2B | 0.0058 |
| chr13 | 168 | No genes in this region | 0.0058 |
| chr1 | 805 | DDR2,C1orf110,HSD17B7 | 0.0059 |
| chr1 | 1198 | FH,KMO | 0.0060 |
| chr5 | 691 | LRRTM2,SIL1,CTNNA1 | 0.0061 |
| chr4 | 788 | No genes in this region | 0.0061 |
| chr5 | 86 | BASP1 | 0.0062 |
| chr11 | 429 | CCDC81,ME3 | 0.0063 |
| chr2 | 897 | CCDC141 | 0.0064 |
| chr2 | 84 | No genes in this region | 0.0064 |
| chr3 | 850 | C3orf50 | 0.0065 |
| chr1 | 295 | JUN | 0.0066 |
| chr15 | 436 | HAPLN3,ACAN,MFGE8 | 0.0067 |
| chr20 | 286 | ZNF831,EDN3 | 0.0067 |
| chr17 | 75 | PMP22,TEKT3 | 0.0068 |
| chr6 | 607 | C6orf170 | 0.0069 |
| chr16 | 53 | FAM18A,TEKT5,NUBP1 | 0.0070 |
| chr12 | 311 | DPY19L2 | 0.0070 |
| chr11 | 399 | No genes in this region | 0.0071 |
| chr11 | 117 | No genes in this region | 0.0072 |
| chr10 | 402 | ZMIZ1,LOC283050 | 0.0073 |
| chr5 | 751 | ZNF300,TNIP1,GPX3 | 0.0073 |
| chr1 | 1024 | MAPKAPK2,RASSF5,DYRK3,LGTN | 0.0074 |
| chr7 | 43 | NXPH1 | 0.0075 |
| chr5 | 168 | ADAMTS12 | 0.0076 |
| chr1 | 1071 | USH2A | 0.0076 |
| chr11 | 473 | SESN3 | 0.0077 |
| chr13 | 131 | GPR12 | 0.0078 |
| chr2 | 685 | No genes in this region | 0.0079 |
| chr3 | 436 | CHMP2B,POU1F1 | 0.0079 |
| chr3 | 852 | MDS1 | 0.0080 |
| chr2 | 885 | No genes in this region | 0.0081 |
| chr12 | 69 | GRIN2B | 0.0082 |
| chr1 | 1048 | RD3,TRAF5 | 0.0082 |
| chr1 | 992 | FAM58B,NR5A2 | 0.0083 |
| chr7 | 378 | HSPB1,YWHAG,FLJ37078 | 0.0084 |
| chr3 | 967 | FGF12 | 0.0085 |
| chr8 | 639 | No genes in this region | 0.0085 |
| chr16 | 133 | No genes in this region | 0.0086 |
| chr3 | 708 | CLSTN2 | 0.0087 |
| chr13 | 238 | ITM2B,RB1 | 0.0088 |
| chr7 | 377 | TMEM120A,MDH2,SNORA14A,STYXL1,POR | 0.0088 |
| chr2 | 519 | No genes in this region | 0.0089 |
| chr5 | 68 | DNAH5 | 0.0090 |
| chr4 | 142 | No genes in this region | 0.0091 |
| chr4 | 113 | No genes in this region | 0.0091 |
| chr20 | 288 | PHACTR3 | 0.0092 |
| chr15 | 232 | FBN1,DUT | 0.0093 |
| chr5 | 7 | SLC6A3,LPCAT1 | 0.0094 |
| chr7 | 516 | RELN | 0.0094 |
| chr8 | 657 | ASAP1 | 0.0095 |
| chr14 | 218 | No genes in this region | 0.0096 |
| chr4 | 308 | No genes in this region | 0.0097 |
| chr7 | 148 | LOC646762 | 0.0097 |
| chr15 | 200 | PLA2G4E,EHD4,PLA2G4D | 0.0098 |
| chr3 | 624 | MYLK | 0.0099 |
| chr1 | 211 | GUCA2B | 0.0100 |
| chr1 | 68 | PRAMEF21,PDPN,PRAMEF20 | 0.0100 |
| chr7 | 348 | AUTS2 | 0.0101 |
| chr2 | 672 | No genes in this region | 0.0102 |
| chr11 | 400 | No genes in this region | 0.0103 |
| chr2 | 18 | ALLC,RPS7,COLEC11 | 0.0103 |
| chr3 | 10 | CNTN4 | 0.0104 |
| chr6 | 845 | No genes in this region | 0.0105 |
| chr15 | 352 | ARIH1,HEXA,TMEM202 | 0.0106 |
| chr5 | 149 | No genes in this region | 0.0106 |
| chr3 | 975 | No genes in this region | 0.0107 |
| chr5 | 733 | STK32A,DPYSL3 | 0.0108 |
| chr13 | 348 | ATXN8OS | 0.0109 |
| chr2 | 1159 | ARMC9,B3GNT7 | 0.0109 |
| chr6 | 149 | HCG4,HLA-F,HLA-G,HCG2P7,IFITM4P,HLA-H | 0.0110 |
| chr15 | 351 | BRUNOL6,PARP6,PKM2,SENP8,GRAMD2 | 0.0111 |
| chr3 | 9 | No genes in this region | 0.0112 |
| chr18 | 99 | TTC39C,CABYR,OSBPL1A | 0.0112 |
| chr4 | 34 | KIAA0232,TBC1D14 | 0.0113 |
| chr20 | 255 | TSHZ2 | 0.0114 |
| chr17 | 119 | SDF2,ALDOC,SLC13A2,FOXN1,FLJ25006,KIAA0100,PIGS,UNC119,SPAG5,LOC645851 | 0.0115 |
| chr17 | 95 | LOC400581,B9D1,EPN2 | 0.0115 |
| chr8 | 481 | C8orf37,PLEKHF2 | 0.0116 |
| chr2 | 300 | No genes in this region | 0.0117 |
| chr2 | 859 | METTL8,C2orf37 | 0.0118 |
| chr3 | 30 | No genes in this region | 0.0118 |
| chr2 | 555 | RGPD7,RGPD5,BUB1 | 0.0119 |
| chr7 | 759 | XRCC2 | 0.0120 |
| chr2 | 855 | MYO3B | 0.0121 |
| chr6 | 14 | FAM136B,SERPINB6,NQO2,SERPINB9 | 0.0121 |
| chr7 | 66 | No genes in this region | 0.0122 |
| chr1 | 421 | PRKACB,TTLL7 | 0.0123 |
| chr22 | 162 | LARGE | 0.0124 |
| chr9 | 67 | No genes in this region | 0.0124 |
| chr1 | 235 | CYP4Z2P,CYP4B1,CYP4A11 | 0.0125 |
| chr18 | 44 | KIAA0802 | 0.0126 |
| chr13 | 167 | RFC3 | 0.0127 |
| chr8 | 265 | ST18 | 0.0127 |
| chr15 | 126 | GABRG3 | 0.0128 |
| chr6 | 608 | GJA1,C6orf170 | 0.0129 |
| chr22 | 196 | MKL1 | 0.0130 |
| chr9 | 377 | No genes in this region | 0.0130 |
| chr13 | 237 | SUCLA2,NUDT15,MED4 | 0.0131 |
| chr11 | 250 | LOC441601 | 0.0132 |
| chr10 | 365 | CDH23,C10orf54 | 0.0133 |
| chr6 | 5 | No genes in this region | 0.0133 |
| chr13 | 152 | C13orf26 | 0.0134 |
| chr2 | 70 | No genes in this region | 0.0135 |
| chr20 | 69 | SEL1L2,MACROD2 | 0.0136 |
| chr4 | 196 | C4orf34,UGDH,UBE2K | 0.0136 |
| chr14 | 177 | BRMS1L | 0.0137 |
| chr15 | 405 | RPS17,AP3B2,CPEB1 | 0.0138 |
| chr1 | 1209 | AKT3 | 0.0139 |
| chr2 | 799 | WDSUB1,BAZ2B | 0.0139 |
| chr13 | 427 | No genes in this region | 0.0140 |
| chr3 | 705 | CLSTN2 | 0.0141 |
| chr8 | 586 | No genes in this region | 0.0142 |
| chr21 | 196 | No genes in this region | 0.0142 |
| chr6 | 381 | SENP6,FILIP1 | 0.0143 |
| chr8 | 32 | AGPAT5,MCPH1,ANGPT2 | 0.0144 |
| chr20 | 95 | LOC100130264,SLC24A3 | 0.0145 |
| chr15 | 239 | ATP8B4 | 0.0145 |
| chr22 | 195 | ADSL,TNRC6B,SGSM3,MKL1 | 0.0146 |
| chr18 | 315 | No genes in this region | 0.0147 |
| chr15 | 425 | AGBL1 | 0.0148 |
| chr1 | 721 | ITGA10,ANKRD35,RBM8A,PEX11B,GNRHR2,LIX1L,RNF115,PIAS3,NUDT17,POLR3C | 0.0148 |
| chr14 | 292 | No genes in this region | 0.0149 |
| chr1 | 1174 | ACTN2,HEATR1 | 0.0150 |
| chr3 | 54 | SLC6A11 | 0.0151 |
| chr2 | 856 | GAD1,MYO3B,SP5 | 0.0151 |
| chr15 | 136 | KIAA0574,NDNL2 | 0.0152 |
| chr22 | 203 | SREBF2,LOC339674,CENPM,NAGA,SEPT3,TNFRSF13C,WBP2NL | 0.0153 |
| chr11 | 660 | OPCML | 0.0154 |
| chr1 | 310 | INADL | 0.0154 |
| chr7 | 111 | RAPGEF5 | 0.0155 |
| chr8 | 619 | ZHX2 | 0.0156 |
| chr16 | 41 | No genes in this region | 0.0157 |
| chr15 | 234 | CEP152,EID1,SHC4 | 0.0157 |
| chr20 | 209 | TOX2 | 0.0158 |
| chr13 | 197 | No genes in this region | 0.0159 |
| chr6 | 693 | KIAA1244,HEBP2 | 0.0160 |
| chr10 | 190 | ZNF248 | 0.0160 |
| chr7 | 330 | TYW1,C7orf42,SBDS | 0.0161 |
| chr6 | 497 | FBXL4 | 0.0162 |
| chr10 | 192 | LOC100129055,ZNF37A | 0.0162 |
| chr15 | 446 | RCCD1,MAN2A2,UNC45A,FURIN,FES,HDDC3,VPS33B,PRC1 | 0.0163 |
| chr14 | 420 | No genes in this region | 0.0164 |
| chr10 | 182 | No genes in this region | 0.0165 |
| chr5 | 166 | No genes in this region | 0.0165 |
| chr4 | 481 | UNC5C,BMPR1B | 0.0166 |
| chr5 | 818 | No genes in this region | 0.0167 |
| chr12 | 312 | TMEM5,SRGAP1 | 0.0168 |
| chr22 | 104 | VPREB1 | 0.0168 |
| chr11 | 200 | LRRC4C | 0.0169 |
| chr9 | 11 | No genes in this region | 0.0170 |
| chr4 | 355 | C4orf40,CSN3,ODAM,CSN1S2B,C4orf7 | 0.0171 |
| chr12 | 600 | CAMKK2,P2RX7,P2RX4 | 0.0171 |
| chr2 | 510 | IL1R2,IL1RL2,IL1R1 | 0.0172 |
| chr17 | 152 | RFFL,AMAC1,SLFN5,FNDC8,UNC45B,NLE1,RAD51L3 | 0.0173 |
| chr10 | 454 | LIPA,CH25H | 0.0174 |
| chr9 | 124 | No genes in this region | 0.0174 |
| chr22 | 89 | CDC45L,C22orf39,UFD1L,LOC150185,CLDN5,MRPL40 | 0.0175 |
| chr10 | 54 | No genes in this region | 0.0176 |
| chr12 | 19 | PARP11 | 0.0177 |
| chr9 | 679 | NCRNA00094,WDR5,BRD3,VAV2 | 0.0177 |
| chr2 | 1124 | FAM124B | 0.0178 |
| chr2 | 847 | SPC25,NOSTRIN,ABCB11,G6PC2 | 0.0179 |
| chr2 | 1167 | NGEF,GIGYF2,UNQ830 | 0.0180 |
| chr17 | 48 | GAS7,GLP2R,DHRS7C,RCVRN | 0.0180 |
| chr8 | 52 | C8orf74,UNQ9391,RP1L1 | 0.0181 |
| chr13 | 198 | No genes in this region | 0.0182 |
| chr10 | 272 | No genes in this region | 0.0183 |
| chr22 | 202 | CCDC134,SREBF2,NHP2L1,MEI1,FLJ23584 | 0.0183 |
| chr7 | 466 | TFPI2,GNG11,GNGT1 | 0.0184 |
| chr12 | 164 | PKP2,YARS2 | 0.0185 |
| chr7 | 40 | GLCCI1,ICA1 | 0.0186 |
| chr10 | 40 | GATA3,FLJ45983,TAF3 | 0.0186 |
| chr3 | 853 | MDS1 | 0.0187 |
| chr13 | 232 | No genes in this region | 0.0188 |
| chr16 | 386 | WWOX | 0.0189 |
| chr13 | 405 | No genes in this region | 0.0189 |
| chr18 | 259 | No genes in this region | 0.0190 |
| chr12 | 437 | KITLG | 0.0191 |
| chr9 | 413 | No genes in this region | 0.0192 |
| chr16 | 108 | OTOA,LOC100190986,LOC730092 | 0.0192 |
| chr12 | 632 | No genes in this region | 0.0193 |
| chr6 | 403 | ELOVL4,TTK | 0.0194 |
| chr15 | 270 | NEDD4,RFX7 | 0.0195 |
| chr11 | 359 | CLPB,PDE2A | 0.0195 |
| chr5 | 263 | No genes in this region | 0.0196 |
| chr19 | 305 | NLRP11,NLRP13,NLRP4,NLRP8 | 0.0197 |
| chr1 | 984 | PTPRC | 0.0198 |
| chr1 | 1210 | AKT3 | 0.0198 |
| chr15 | 350 | SENP8,MYO9A | 0.0199 |
| chr9 | 127 | No genes in this region | 0.0200 |
