# Supplementary material for: Genomic Analysis of Natural Selection and Phenotypic Variation in High-Altitude Mongolians
Source: PLoS Genet. 2013 Jul 18;9(7):e1003634. doi: 10.1371/journal.pgen.1003634 (PMC3715426; doi:10.1371/journal.pgen.1003634)
Supplement: Table S4 — Top 2% of selection candidate genes identified in the MR analysis in DU Mongolians. (DOCX) [file pgen.1003634.s005.docx]

**Table S4. Top 2% of selection candidate genes identified in the MR analysis in DU Mongolians**

| **Gene** | **Chr** | **Begin** | **End** | **nSNP** | **nTail SNPs 05** | **nTail SNPs 01** | **nTail SNPs 005** | **MR05** | **MR01** | **MR005** | **P05** | **P01** | **P005** |
| --- | --- | --- | --- | --- | --- | --- | --- | --- | --- | --- | --- | --- | --- |
| CUL2 | chr10 | 35328811 | 35429300 | 12 | 11 | 7 | 0 | 13.91 | 25.8 | 0 | 1 | 0.998 | 0 |
| GFPT1 | chr2 | 69390408 | 69477886 | 13 | 11 | 0 | 0 | 12.84 | 0 | 0 | 1 | 0 | 0 |
| KISS1 | chr1 | 202416091 | 202442242 | 11 | 9 | 6 | 1 | 12.42 | 24.12 | 6.32 | 1 | 0.998 | 0.948 |
| LCE1E | chr1 | 151015376 | 151037525 | 11 | 9 | 6 | 4 | 12.42 | 24.12 | 25.27 | 1 | 0.998 | 0.995 |
| NET1 | chr10 | 5434517 | 5500426 | 16 | 13 | 13 | 13 | 12.33 | 35.93 | 56.47 | 0.999 | 1 | 1 |
| VPS13C | chr15 | 59921881 | 60149939 | 24 | 19 | 12 | 10 | 12.02 | 22.11 | 28.96 | 0.999 | 0.997 | 0.997 |
| GLIPR1L2 | chr12 | 74061155 | 74114087 | 14 | 11 | 10 | 10 | 11.93 | 31.59 | 49.64 | 0.999 | 1 | 1 |
| KLK8 | chr19 | 56181075 | 56206770 | 12 | 9 | 5 | 5 | 11.38 | 18.43 | 28.96 | 0.999 | 0.995 | 0.997 |
| LCE1F | chr1 | 151005471 | 151025828 | 12 | 9 | 6 | 4 | 11.38 | 22.11 | 23.17 | 0.999 | 0.997 | 0.992 |
| XRN2 | chr20 | 21221941 | 21328463 | 16 | 12 | 11 | 8 | 11.38 | 30.4 | 34.75 | 0.999 | 0.999 | 0.998 |
| LOC92973 | chr9 | 35840270 | 35865515 | 11 | 8 | 3 | 3 | 11.04 | 12.06 | 18.95 | 0.998 | 0.982 | 0.989 |
| NDUFS2 | chr1 | 159425728 | 159460808 | 11 | 8 | 2 | 0 | 11.04 | 8.04 | 0 | 0.998 | 0.962 | 0 |
| OR5B3 | chr11 | 57916513 | 57937458 | 11 | 8 | 8 | 8 | 11.04 | 32.16 | 50.55 | 0.998 | 1 | 1 |
| SLC30A6 | chr2 | 32234436 | 32310313 | 11 | 8 | 8 | 3 | 11.04 | 32.16 | 18.95 | 0.998 | 1 | 0.989 |
| SYCE1 | chr10 | 135207394 | 135242866 | 22 | 16 | 15 | 3 | 11.04 | 30.15 | 9.48 | 0.998 | 0.999 | 0.969 |
| ZNF148 | chr3 | 126417202 | 126586888 | 31 | 22 | 1 | 0 | 10.77 | 1.43 | 0 | 0.998 | 0.819 | 0 |
| BLVRA | chr7 | 43754796 | 43823466 | 10 | 7 | 3 | 2 | 10.62 | 13.27 | 13.9 | 0.998 | 0.986 | 0.981 |
| IFNGR2 | chr21 | 33687071 | 33741698 | 10 | 7 | 5 | 3 | 10.62 | 22.11 | 20.85 | 0.998 | 0.997 | 0.991 |
| ST3GAL5 | chr2 | 85909781 | 85979668 | 20 | 14 | 11 | 11 | 10.62 | 24.32 | 38.23 | 0.998 | 0.998 | 0.999 |
| C16orf73 | chr16 | 1813989 | 1872103 | 22 | 15 | 6 | 0 | 10.35 | 12.06 | 0 | 0.998 | 0.982 | 0 |
| C12orf51 | chr12 | 111072493 | 111238421 | 21 | 14 | 13 | 13 | 10.12 | 27.38 | 43.02 | 0.997 | 0.999 | 0.999 |
| CD46 | chr1 | 205982024 | 206045481 | 12 | 8 | 7 | 4 | 10.12 | 25.8 | 23.17 | 0.997 | 0.998 | 0.992 |
| CDC16 | chr13 | 114008463 | 114066252 | 12 | 8 | 3 | 1 | 10.12 | 11.06 | 5.79 | 0.997 | 0.978 | 0.942 |
| GRK4 | chr4 | 2925140 | 3022272 | 27 | 18 | 4 | 3 | 10.12 | 6.55 | 7.72 | 0.997 | 0.951 | 0.963 |
| IPO7 | chr11 | 9352779 | 9433651 | 15 | 10 | 10 | 10 | 10.12 | 29.48 | 46.33 | 0.997 | 0.999 | 1 |
| MDH2 | chr7 | 75505328 | 75543866 | 12 | 8 | 7 | 4 | 10.12 | 25.8 | 23.17 | 0.997 | 0.998 | 0.992 |
| SLC28A2 | chr15 | 43321725 | 43365425 | 24 | 16 | 0 | 0 | 10.12 | 0 | 0 | 0.997 | 0 | 0 |
| SFRS2B | chr11 | 94429703 | 94454036 | 14 | 9 | 3 | 2 | 9.76 | 9.48 | 9.93 | 0.997 | 0.973 | 0.97 |
| TRPC2 | chr11 | 3594289 | 3625365 | 14 | 9 | 5 | 4 | 9.76 | 15.79 | 19.86 | 0.997 | 0.99 | 0.99 |
| AFF4 | chr5 | 132228969 | 132337253 | 11 | 7 | 4 | 4 | 9.66 | 16.08 | 25.27 | 0.996 | 0.991 | 0.995 |
| C4orf10 | chr4 | 2897075 | 2932591 | 11 | 7 | 1 | 0 | 9.66 | 4.02 | 0 | 0.996 | 0.915 | 0 |
| CPOX | chr3 | 99770979 | 99805131 | 11 | 7 | 4 | 2 | 9.66 | 16.08 | 12.64 | 0.996 | 0.991 | 0.978 |
| KIAA1377 | chr11 | 101280955 | 101387003 | 22 | 14 | 0 | 0 | 9.66 | 0 | 0 | 0.996 | 0 | 0 |
| SIM1 | chr6 | 100933470 | 101028272 | 33 | 21 | 16 | 16 | 9.66 | 21.44 | 33.7 | 0.996 | 0.997 | 0.998 |
| SR140 | chr3 | 144193061 | 144272257 | 22 | 14 | 2 | 1 | 9.66 | 4.02 | 3.16 | 0.996 | 0.915 | 0.902 |
| SSTR4 | chr20 | 22954056 | 22975314 | 11 | 7 | 4 | 3 | 9.66 | 16.08 | 18.95 | 0.996 | 0.991 | 0.989 |
| TFAP2D | chr6 | 50779215 | 50858705 | 11 | 7 | 7 | 4 | 9.66 | 28.14 | 25.27 | 0.996 | 0.999 | 0.995 |
| ZNF37A | chr10 | 38413280 | 38462284 | 11 | 7 | 1 | 1 | 9.66 | 4.02 | 6.32 | 0.996 | 0.915 | 0.948 |
| MCCC2 | chr5 | 70908870 | 71000286 | 24 | 15 | 4 | 2 | 9.49 | 7.37 | 5.79 | 0.996 | 0.956 | 0.942 |
| TUBAL3 | chr10 | 5415060 | 5446793 | 16 | 10 | 10 | 10 | 9.49 | 27.64 | 43.44 | 0.996 | 0.999 | 0.999 |
| GOLT1A | chr1 | 202423910 | 202459843 | 13 | 8 | 5 | 0 | 9.34 | 17.01 | 0 | 0.996 | 0.993 | 0 |
| NGDN | chr14 | 22998737 | 23027242 | 13 | 8 | 3 | 0 | 9.34 | 10.21 | 0 | 0.996 | 0.975 | 0 |
| TFPI2 | chr7 | 93343680 | 93368001 | 13 | 8 | 7 | 7 | 9.34 | 23.81 | 37.42 | 0.996 | 0.998 | 0.999 |
| GJB6 | chr13 | 19684100 | 19714534 | 10 | 6 | 2 | 1 | 9.11 | 8.84 | 6.95 | 0.995 | 0.967 | 0.955 |
| GOLPH3L | chr1 | 148875324 | 148946296 | 20 | 12 | 10 | 3 | 9.11 | 22.11 | 10.43 | 0.995 | 0.997 | 0.972 |
| GOSR2 | chr17 | 42345484 | 42383732 | 20 | 12 | 2 | 1 | 9.11 | 4.42 | 3.48 | 0.995 | 0.925 | 0.907 |
| HUS1 | chr7 | 47960307 | 47995771 | 10 | 6 | 5 | 5 | 9.11 | 22.11 | 34.75 | 0.995 | 0.997 | 0.998 |
| NOP14 | chr4 | 2899461 | 2944916 | 10 | 6 | 1 | 0 | 9.11 | 4.42 | 0 | 0.995 | 0.925 | 0 |
| TAS2R49 | chr12 | 11029827 | 11051741 | 10 | 6 | 0 | 0 | 9.11 | 0 | 0 | 0.995 | 0 | 0 |
| ZNF554 | chr19 | 2760871 | 2797733 | 10 | 6 | 6 | 5 | 9.11 | 26.53 | 34.75 | 0.995 | 0.999 | 0.998 |
| ZNF555 | chr19 | 2782481 | 2815035 | 10 | 6 | 6 | 5 | 9.11 | 26.53 | 34.75 | 0.995 | 0.999 | 0.998 |
| DIXDC1 | chr11 | 111303136 | 111408517 | 12 | 7 | 2 | 0 | 8.85 | 7.37 | 0 | 0.994 | 0.956 | 0 |
| KPNA5 | chr6 | 117099059 | 117179723 | 12 | 7 | 6 | 6 | 8.85 | 22.11 | 34.75 | 0.994 | 0.997 | 0.998 |
| ZNF787 | chr19 | 61280543 | 61334461 | 12 | 7 | 5 | 0 | 8.85 | 18.43 | 0 | 0.994 | 0.995 | 0 |
| ZNF829 | chr19 | 42064092 | 42109030 | 12 | 7 | 2 | 0 | 8.85 | 7.37 | 0 | 0.994 | 0.956 | 0 |
| BDP1 | chr5 | 70777197 | 70909405 | 26 | 15 | 1 | 0 | 8.76 | 1.7 | 0 | 0.994 | 0.833 | 0 |
| DEPDC1B | chr5 | 59918495 | 60041713 | 14 | 8 | 5 | 5 | 8.67 | 15.79 | 24.82 | 0.994 | 0.99 | 0.994 |
| GLUL | chr1 | 180608291 | 180637573 | 14 | 8 | 0 | 0 | 8.67 | 0 | 0 | 0.994 | 0 | 0 |
| GOLSYN | chr8 | 110645580 | 110783196 | 42 | 24 | 19 | 14 | 8.67 | 20.01 | 23.17 | 0.994 | 0.996 | 0.992 |
| RNF212 | chr4 | 1045268 | 1107582 | 21 | 12 | 4 | 0 | 8.67 | 8.42 | 0 | 0.994 | 0.966 | 0 |
| RWDD1 | chr6 | 116989275 | 117031129 | 14 | 8 | 8 | 8 | 8.67 | 25.27 | 39.71 | 0.994 | 0.998 | 0.999 |
| TEDDM1 | chr1 | 180623874 | 180646374 | 14 | 8 | 1 | 1 | 8.67 | 3.16 | 4.96 | 0.994 | 0.892 | 0.931 |
| FOXJ3 | chr1 | 42404796 | 42583490 | 23 | 13 | 2 | 0 | 8.58 | 3.85 | 0 | 0.994 | 0.912 | 0 |
| HICE1 | chr19 | 17011570 | 17057343 | 16 | 9 | 2 | 1 | 8.54 | 5.53 | 4.34 | 0.993 | 0.941 | 0.921 |
| UMPS | chr3 | 125921902 | 125956730 | 16 | 9 | 4 | 0 | 8.54 | 11.06 | 0 | 0.993 | 0.978 | 0 |
| FDX1 | chr11 | 109795803 | 109850815 | 27 | 15 | 9 | 9 | 8.43 | 14.74 | 23.17 | 0.993 | 0.988 | 0.992 |
| GBF1 | chr10 | 103985298 | 104142639 | 18 | 10 | 1 | 1 | 8.43 | 2.46 | 3.86 | 0.993 | 0.866 | 0.913 |
| GSTA4 | chr6 | 52940705 | 52978137 | 18 | 10 | 9 | 8 | 8.43 | 22.11 | 30.89 | 0.993 | 0.997 | 0.998 |
| FRRS1 | chr1 | 99936846 | 100013937 | 31 | 17 | 14 | 6 | 8.32 | 19.97 | 13.45 | 0.993 | 0.996 | 0.981 |
| ARNT | chr1 | 149038809 | 149125810 | 11 | 6 | 3 | 2 | 8.28 | 12.06 | 12.64 | 0.991 | 0.982 | 0.978 |
| C12orf30 | chr12 | 110938875 | 111040980 | 11 | 6 | 5 | 5 | 8.28 | 20.1 | 31.59 | 0.991 | 0.996 | 0.998 |
| C15orf42 | chr15 | 87909821 | 87982257 | 22 | 12 | 0 | 0 | 8.28 | 0 | 0 | 0.991 | 0 | 0 |
| CCL4 | chr17 | 31445332 | 31467127 | 11 | 6 | 4 | 3 | 8.28 | 16.08 | 18.95 | 0.991 | 0.991 | 0.989 |
| CDRT4 | chr17 | 15270062 | 15321650 | 22 | 12 | 4 | 4 | 8.28 | 8.04 | 12.64 | 0.991 | 0.962 | 0.978 |
| FAM55C | chr3 | 102970718 | 103039763 | 11 | 6 | 1 | 1 | 8.28 | 4.02 | 6.32 | 0.991 | 0.915 | 0.948 |
| HMSD | chr18 | 59757567 | 59788625 | 11 | 6 | 0 | 0 | 8.28 | 0 | 0 | 0.991 | 0 | 0 |
| LOC285194 | chr3 | 117901324 | 117928575 | 22 | 12 | 3 | 3 | 8.28 | 6.03 | 9.48 | 0.991 | 0.947 | 0.969 |
| NSL1 | chr1 | 210956117 | 211041762 | 22 | 12 | 0 | 0 | 8.28 | 0 | 0 | 0.991 | 0 | 0 |
| OR1S1 | chr11 | 57728792 | 57749770 | 11 | 6 | 2 | 1 | 8.28 | 8.04 | 6.32 | 0.991 | 0.962 | 0.948 |
| RBP5 | chr12 | 7157553 | 7182733 | 11 | 6 | 1 | 0 | 8.28 | 4.02 | 0 | 0.991 | 0.915 | 0 |
| TCL1B | chr14 | 95212515 | 95238720 | 11 | 6 | 4 | 4 | 8.28 | 16.08 | 25.27 | 0.991 | 0.991 | 0.995 |
| TMEM155 | chr4 | 122889534 | 122915790 | 11 | 6 | 4 | 3 | 8.28 | 16.08 | 18.95 | 0.991 | 0.991 | 0.989 |
| VAMP8 | chr2 | 85648158 | 85672667 | 11 | 6 | 6 | 6 | 8.28 | 24.12 | 37.91 | 0.991 | 0.998 | 0.999 |
| ZNF143 | chr11 | 9429088 | 9516647 | 11 | 6 | 5 | 5 | 8.28 | 20.1 | 31.59 | 0.991 | 0.996 | 0.998 |
| MKL1 | chr22 | 39126237 | 39372636 | 24 | 13 | 8 | 8 | 8.22 | 14.74 | 23.17 | 0.991 | 0.988 | 0.992 |
| HLA-G | chr6 | 29892734 | 29916878 | 13 | 7 | 4 | 3 | 8.17 | 13.61 | 16.04 | 0.991 | 0.987 | 0.986 |
| OR4B1 | chr11 | 48184937 | 48205867 | 13 | 7 | 4 | 2 | 8.17 | 13.61 | 10.69 | 0.991 | 0.987 | 0.973 |
| SRD5A2 | chr2 | 31593159 | 31669544 | 13 | 7 | 4 | 4 | 8.17 | 13.61 | 21.38 | 0.991 | 0.987 | 0.991 |
| UBE2D3 | chr4 | 103926216 | 104019473 | 13 | 7 | 0 | 0 | 8.17 | 0 | 0 | 0.991 | 0 | 0 |
| VAMP5 | chr2 | 85655041 | 85684022 | 13 | 7 | 6 | 6 | 8.17 | 20.41 | 32.08 | 0.991 | 0.996 | 0.998 |
| C11orf49 | chr11 | 46904826 | 47152507 | 28 | 15 | 2 | 2 | 8.13 | 3.16 | 4.96 | 0.991 | 0.892 | 0.931 |
| ADPGK | chr15 | 70820760 | 70873179 | 15 | 8 | 6 | 6 | 8.09 | 17.69 | 27.8 | 0.99 | 0.994 | 0.997 |
| C9orf127 | chr9 | 35809221 | 35854844 | 15 | 8 | 2 | 2 | 8.09 | 5.9 | 9.27 | 0.99 | 0.945 | 0.968 |
| CLIP2 | chr7 | 73331740 | 73468209 | 15 | 8 | 5 | 5 | 8.09 | 14.74 | 23.17 | 0.99 | 0.988 | 0.992 |
| ITCH | chr20 | 32404722 | 32572858 | 15 | 8 | 2 | 0 | 8.09 | 5.9 | 0 | 0.99 | 0.945 | 0 |
| FLYWCH1 | chr16 | 2891980 | 2951210 | 17 | 9 | 7 | 7 | 8.04 | 18.21 | 28.62 | 0.99 | 0.995 | 0.997 |
| MYO18A | chr17 | 24414653 | 24541533 | 17 | 9 | 5 | 0 | 8.04 | 13.01 | 0 | 0.99 | 0.985 | 0 |
| TDH | chr8 | 11224555 | 11273371 | 17 | 9 | 4 | 2 | 8.04 | 10.41 | 8.18 | 0.99 | 0.976 | 0.964 |
| DHCR7 | chr11 | 70813104 | 70847125 | 19 | 10 | 9 | 8 | 7.99 | 20.95 | 29.26 | 0.99 | 0.997 | 0.997 |
| LCMT1 | chr16 | 25020547 | 25107052 | 19 | 10 | 4 | 1 | 7.99 | 9.31 | 3.66 | 0.99 | 0.972 | 0.91 |
| SREBF2 | chr22 | 40549051 | 40642321 | 19 | 10 | 7 | 6 | 7.99 | 16.29 | 21.95 | 0.99 | 0.993 | 0.992 |
| CPEB1 | chr15 | 80999005 | 81123783 | 23 | 12 | 12 | 10 | 7.92 | 23.07 | 30.22 | 0.99 | 0.998 | 0.998 |
| OAS3 | chr12 | 111850631 | 111905438 | 27 | 14 | 8 | 6 | 7.87 | 13.1 | 15.44 | 0.989 | 0.985 | 0.985 |
| SPSB4 | chr3 | 142243432 | 142360143 | 27 | 14 | 5 | 4 | 7.87 | 8.19 | 10.3 | 0.989 | 0.965 | 0.972 |
| SFRS8 | chr12 | 130751587 | 130860235 | 29 | 15 | 9 | 6 | 7.85 | 13.72 | 14.38 | 0.989 | 0.988 | 0.983 |
| NOS2 | chr17 | 23097919 | 23161682 | 31 | 16 | 11 | 9 | 7.83 | 15.69 | 20.18 | 0.989 | 0.99 | 0.991 |
| C9orf91 | chr9 | 116403526 | 116458524 | 33 | 17 | 2 | 0 | 7.82 | 2.68 | 0 | 0.989 | 0.876 | 0 |
| POLN | chr4 | 2033442 | 2210756 | 35 | 18 | 2 | 1 | 7.81 | 2.53 | 1.99 | 0.989 | 0.871 | 0.878 |
| DOCK3 | chr3 | 50677675 | 51406669 | 74 | 38 | 26 | 3 | 7.79 | 15.54 | 2.82 | 0.989 | 0.99 | 0.897 |
| ART1 | chr11 | 3612936 | 3652222 | 16 | 8 | 8 | 6 | 7.59 | 22.11 | 26.06 | 0.986 | 0.997 | 0.996 |
| ATF7IP2 | chr16 | 10420225 | 10494996 | 24 | 12 | 0 | 0 | 7.59 | 0 | 0 | 0.986 | 0 | 0 |
| C1orf201 | chr1 | 24546075 | 24610536 | 18 | 9 | 7 | 7 | 7.59 | 17.2 | 27.03 | 0.986 | 0.994 | 0.996 |
| CCDC15 | chr11 | 124319226 | 124426595 | 16 | 8 | 1 | 1 | 7.59 | 2.76 | 4.34 | 0.986 | 0.878 | 0.921 |
| CLEC2A | chr12 | 9947092 | 9986247 | 22 | 11 | 3 | 3 | 7.59 | 6.03 | 9.48 | 0.986 | 0.947 | 0.969 |
| DIO2 | chr14 | 79723621 | 79758278 | 10 | 5 | 2 | 1 | 7.59 | 8.84 | 6.95 | 0.986 | 0.967 | 0.955 |
| FBXL4 | chr6 | 99418321 | 99512570 | 22 | 11 | 7 | 7 | 7.59 | 14.07 | 22.11 | 0.986 | 0.988 | 0.992 |
| GARNL1 | chr14 | 35067308 | 35358183 | 28 | 14 | 9 | 8 | 7.59 | 14.21 | 19.86 | 0.986 | 0.988 | 0.99 |
| GP5 | chr3 | 195586838 | 195611284 | 10 | 5 | 1 | 1 | 7.59 | 4.42 | 6.95 | 0.986 | 0.925 | 0.955 |
| KLK7 | chr19 | 56161540 | 56188962 | 12 | 6 | 4 | 4 | 7.59 | 14.74 | 23.17 | 0.986 | 0.988 | 0.992 |
| LASS5 | chr12 | 48799847 | 48857364 | 12 | 6 | 0 | 0 | 7.59 | 0 | 0 | 0.986 | 0 | 0 |
| LOC100129055 | chr10 | 38494604 | 38553278 | 12 | 6 | 4 | 0 | 7.59 | 14.74 | 0 | 0.986 | 0.988 | 0 |
| LOC100192379 | chr4 | 122895189 | 122917412 | 12 | 6 | 3 | 3 | 7.59 | 11.06 | 17.38 | 0.986 | 0.978 | 0.987 |
| LPXN | chr11 | 58040919 | 58109910 | 10 | 5 | 1 | 1 | 7.59 | 4.42 | 6.95 | 0.986 | 0.925 | 0.955 |
| LRP11 | chr6 | 150171624 | 150237173 | 16 | 8 | 0 | 0 | 7.59 | 0 | 0 | 0.986 | 0 | 0 |
| MND1 | chr4 | 154475250 | 154565693 | 14 | 7 | 1 | 1 | 7.59 | 3.16 | 4.96 | 0.986 | 0.892 | 0.931 |
| NPAL3 | chr1 | 24604831 | 24682059 | 26 | 13 | 3 | 1 | 7.59 | 5.1 | 2.67 | 0.986 | 0.937 | 0.894 |
| NUP98 | chr11 | 3642815 | 3785468 | 28 | 14 | 14 | 12 | 7.59 | 22.11 | 29.79 | 0.986 | 0.997 | 0.998 |
| PDS5A | chr4 | 39490877 | 39665971 | 20 | 10 | 2 | 0 | 7.59 | 4.42 | 0 | 0.986 | 0.925 | 0 |
| PTGES | chr9 | 131530436 | 131565165 | 12 | 6 | 0 | 0 | 7.59 | 0 | 0 | 0.986 | 0 | 0 |
| SCRN3 | chr2 | 174958712 | 175011974 | 12 | 6 | 0 | 0 | 7.59 | 0 | 0 | 0.986 | 0 | 0 |
| SCUBE3 | chr6 | 35280167 | 35336587 | 10 | 5 | 3 | 1 | 7.59 | 13.27 | 6.95 | 0.986 | 0.986 | 0.955 |
| TMEM63B | chr6 | 44193353 | 44241234 | 12 | 6 | 4 | 4 | 7.59 | 14.74 | 23.17 | 0.986 | 0.988 | 0.992 |
| TOP1P2 | chr22 | 23480467 | 23501986 | 10 | 5 | 2 | 1 | 7.59 | 8.84 | 6.95 | 0.986 | 0.967 | 0.955 |
| WASL | chr7 | 123099232 | 123186352 | 30 | 15 | 5 | 3 | 7.59 | 7.37 | 6.95 | 0.986 | 0.956 | 0.955 |
| ZFC3H1 | chr12 | 70279648 | 70354016 | 16 | 8 | 2 | 1 | 7.59 | 5.53 | 4.34 | 0.986 | 0.941 | 0.921 |
| ZNF345 | chr19 | 42023106 | 42072310 | 10 | 5 | 2 | 1 | 7.59 | 8.84 | 6.95 | 0.986 | 0.967 | 0.955 |
| ZNF420 | chr19 | 42251221 | 42322491 | 12 | 6 | 1 | 1 | 7.59 | 3.69 | 5.79 | 0.986 | 0.907 | 0.942 |
| ZNF558 | chr19 | 8771381 | 8804565 | 12 | 6 | 3 | 2 | 7.59 | 11.06 | 11.58 | 0.986 | 0.978 | 0.976 |
| CNNM2 | chr10 | 104658103 | 104838230 | 35 | 17 | 6 | 0 | 7.37 | 7.58 | 0 | 0.986 | 0.96 | 0 |
| KITLG | chr12 | 87400697 | 87508369 | 31 | 15 | 13 | 11 | 7.34 | 18.55 | 24.66 | 0.986 | 0.995 | 0.994 |
| ABCA6 | chr17 | 64576441 | 64659610 | 29 | 14 | 10 | 9 | 7.33 | 15.25 | 21.57 | 0.986 | 0.99 | 0.992 |
| ADD1 | chr4 | 2805381 | 2911587 | 25 | 12 | 10 | 5 | 7.29 | 17.69 | 13.9 | 0.986 | 0.994 | 0.981 |
| MAPKAPK2 | chr1 | 204914911 | 204984249 | 25 | 12 | 9 | 9 | 7.29 | 15.92 | 25.02 | 0.986 | 0.991 | 0.994 |
| VPS13B | chr8 | 100084669 | 100968984 | 96 | 46 | 16 | 8 | 7.27 | 7.37 | 5.79 | 0.985 | 0.956 | 0.942 |
| BIRC6 | chr2 | 32425599 | 32707469 | 23 | 11 | 9 | 7 | 7.26 | 17.3 | 21.15 | 0.985 | 0.994 | 0.991 |
| OPALIN | chr10 | 98082964 | 98119082 | 23 | 11 | 2 | 0 | 7.26 | 3.85 | 0 | 0.985 | 0.912 | 0 |
| ISCA1L | chr5 | 62096952 | 62118926 | 21 | 10 | 3 | 3 | 7.23 | 6.32 | 9.93 | 0.985 | 0.949 | 0.97 |
| CREM | chr10 | 35445806 | 35551892 | 19 | 9 | 0 | 0 | 7.19 | 0 | 0 | 0.985 | 0 | 0 |
| GATAD2A | chr19 | 19347641 | 19490741 | 19 | 9 | 1 | 0 | 7.19 | 2.33 | 0 | 0.985 | 0.86 | 0 |
| PMFBP1 | chr16 | 70700498 | 70773525 | 19 | 9 | 7 | 7 | 7.19 | 16.29 | 25.61 | 0.985 | 0.993 | 0.996 |
| CCDC64 | chr12 | 118902030 | 119026682 | 17 | 8 | 8 | 8 | 7.14 | 20.81 | 32.71 | 0.984 | 0.997 | 0.998 |
| GPX3 | chr5 | 150370191 | 150398747 | 17 | 8 | 5 | 2 | 7.14 | 13.01 | 8.18 | 0.984 | 0.985 | 0.964 |
| LGTN | chr1 | 204821597 | 204862527 | 17 | 8 | 2 | 1 | 7.14 | 5.2 | 4.09 | 0.984 | 0.938 | 0.917 |
| LOC100130958 | chr16 | 75780849 | 75814477 | 17 | 8 | 0 | 0 | 7.14 | 0 | 0 | 0.984 | 0 | 0 |
| ZNF578 | chr19 | 57638640 | 57721943 | 32 | 15 | 10 | 0 | 7.11 | 13.82 | 0 | 0.984 | 0.988 | 0 |
| GLCCI1 | chr7 | 7964947 | 8105234 | 47 | 22 | 14 | 12 | 7.1 | 13.17 | 17.74 | 0.984 | 0.985 | 0.988 |
| LMNB1 | chr5 | 126130731 | 126210608 | 15 | 7 | 2 | 2 | 7.08 | 5.9 | 9.27 | 0.984 | 0.945 | 0.968 |
| LOC286238 | chr9 | 90441913 | 90466895 | 15 | 7 | 7 | 6 | 7.08 | 20.64 | 27.8 | 0.984 | 0.996 | 0.997 |
| MTMR12 | chr5 | 32252867 | 32358871 | 15 | 7 | 1 | 0 | 7.08 | 2.95 | 0 | 0.984 | 0.885 | 0 |
| RHBDL3 | chr17 | 27607307 | 27685793 | 15 | 7 | 1 | 1 | 7.08 | 2.95 | 4.63 | 0.984 | 0.885 | 0.926 |
| SLFN14 | chr17 | 30889256 | 30919223 | 15 | 7 | 4 | 4 | 7.08 | 11.79 | 18.53 | 0.984 | 0.981 | 0.988 |
| THBD | chr20 | 22964269 | 22988301 | 15 | 7 | 3 | 3 | 7.08 | 8.84 | 13.9 | 0.984 | 0.967 | 0.981 |
| PCNX | chr14 | 70433874 | 70661852 | 28 | 13 | 5 | 1 | 7.05 | 7.9 | 2.48 | 0.983 | 0.961 | 0.89 |
| AASDH | chr4 | 56889213 | 56958395 | 13 | 6 | 0 | 0 | 7.01 | 0 | 0 | 0.982 | 0 | 0 |
| AP4E1 | chr15 | 48978237 | 49095389 | 13 | 6 | 4 | 3 | 7.01 | 13.61 | 16.04 | 0.982 | 0.987 | 0.986 |
| C10orf10 | chr10 | 44781714 | 44804336 | 13 | 6 | 1 | 1 | 7.01 | 3.4 | 5.35 | 0.982 | 0.9 | 0.937 |
| C2orf63 | chr2 | 55243187 | 55323203 | 13 | 6 | 5 | 5 | 7.01 | 17.01 | 26.73 | 0.982 | 0.993 | 0.996 |
| CXCL1 | chr4 | 74943972 | 74965817 | 13 | 6 | 0 | 0 | 7.01 | 0 | 0 | 0.982 | 0 | 0 |
| EPB41L5 | chr2 | 120477138 | 120663165 | 26 | 12 | 1 | 0 | 7.01 | 1.7 | 0 | 0.982 | 0.833 | 0 |
| MAPKAPK5 | chr12 | 110754661 | 110825611 | 13 | 6 | 5 | 5 | 7.01 | 17.01 | 26.73 | 0.982 | 0.993 | 0.996 |
| NR1D2 | chr3 | 23951809 | 24006241 | 13 | 6 | 2 | 2 | 7.01 | 6.8 | 10.69 | 0.982 | 0.953 | 0.973 |
| PPP3R1 | chr2 | 68249492 | 68343155 | 13 | 6 | 4 | 0 | 7.01 | 13.61 | 0 | 0.982 | 0.987 | 0 |
| STK17A | chr7 | 43579216 | 43643503 | 13 | 6 | 1 | 0 | 7.01 | 3.4 | 0 | 0.982 | 0.9 | 0 |
| TYSND1 | chr10 | 71557738 | 71586502 | 13 | 6 | 4 | 3 | 7.01 | 13.61 | 16.04 | 0.982 | 0.987 | 0.986 |
| BANP | chr16 | 86532538 | 86678425 | 50 | 23 | 12 | 10 | 6.98 | 10.61 | 13.9 | 0.982 | 0.977 | 0.981 |
| ACER3 | chr11 | 76239564 | 76422498 | 37 | 17 | 13 | 13 | 6.97 | 15.54 | 24.42 | 0.982 | 0.99 | 0.994 |
| MC2R | chr18 | 13862042 | 13915535 | 24 | 11 | 0 | 0 | 6.96 | 0 | 0 | 0.982 | 0 | 0 |
| GTF2F2 | chr13 | 44582630 | 44766239 | 35 | 16 | 0 | 0 | 6.94 | 0 | 0 | 0.982 | 0 | 0 |
| HAGH | chr16 | 1789104 | 1827196 | 11 | 5 | 1 | 1 | 6.9 | 4.02 | 6.32 | 0.981 | 0.915 | 0.948 |
| KCTD20 | chr6 | 36508521 | 36576293 | 11 | 5 | 0 | 0 | 6.9 | 0 | 0 | 0.981 | 0 | 0 |
| MANBAL | chr20 | 35341464 | 35389077 | 11 | 5 | 0 | 0 | 6.9 | 0 | 0 | 0.981 | 0 | 0 |
| NR1I3 | chr1 | 159456079 | 159484624 | 11 | 5 | 2 | 2 | 6.9 | 8.04 | 12.64 | 0.981 | 0.962 | 0.978 |
| SETD2 | chr3 | 47022903 | 47190471 | 11 | 5 | 5 | 0 | 6.9 | 20.1 | 0 | 0.981 | 0.996 | 0 |
| TIGD2 | chr4 | 90242990 | 90265075 | 11 | 5 | 2 | 0 | 6.9 | 8.04 | 0 | 0.981 | 0.962 | 0 |
| TMEM209 | chr7 | 129581790 | 129642574 | 11 | 5 | 5 | 3 | 6.9 | 20.1 | 18.95 | 0.981 | 0.996 | 0.989 |
| UBE2L6 | chr11 | 57065704 | 57102029 | 11 | 5 | 5 | 5 | 6.9 | 20.1 | 31.59 | 0.981 | 0.996 | 0.998 |
| MYH15 | chr3 | 109571905 | 109740859 | 53 | 24 | 19 | 14 | 6.87 | 15.85 | 18.36 | 0.981 | 0.991 | 0.988 |
| CAPS2 | chr12 | 73946025 | 74020103 | 20 | 9 | 7 | 2 | 6.83 | 15.48 | 6.95 | 0.981 | 0.99 | 0.955 |
| CPA4 | chr7 | 129710229 | 129761250 | 20 | 9 | 4 | 3 | 6.83 | 8.84 | 10.43 | 0.981 | 0.967 | 0.972 |
| PPP2R1A | chr19 | 57375002 | 57431482 | 20 | 9 | 6 | 2 | 6.83 | 13.27 | 6.95 | 0.981 | 0.986 | 0.955 |
| YAF2 | chr12 | 40827173 | 40928317 | 20 | 9 | 6 | 3 | 6.83 | 13.27 | 10.43 | 0.981 | 0.986 | 0.972 |
| PIWIL3 | chr22 | 23435000 | 23510683 | 29 | 13 | 8 | 7 | 6.8 | 12.2 | 16.78 | 0.981 | 0.983 | 0.987 |
| C5orf45 | chr5 | 179186871 | 179228446 | 18 | 8 | 1 | 1 | 6.75 | 2.46 | 3.86 | 0.98 | 0.866 | 0.913 |
| GLCE | chr15 | 67230026 | 67361598 | 27 | 12 | 4 | 0 | 6.75 | 6.55 | 0 | 0.98 | 0.951 | 0 |
| HMHB1 | chr5 | 143161918 | 143190477 | 18 | 8 | 6 | 6 | 6.75 | 14.74 | 23.17 | 0.98 | 0.988 | 0.992 |
| METT5D1 | chr11 | 28076373 | 28321630 | 27 | 12 | 0 | 0 | 6.75 | 0 | 0 | 0.98 | 0 | 0 |
| PBLD | chr10 | 69702422 | 69772690 | 18 | 8 | 0 | 0 | 6.75 | 0 | 0 | 0.98 | 0 | 0 |

MR_Factor: multiple regression factor; P: empirical p-value, aka transformed rank metric; the 05, 01, and 005 suffixes refer to the alpha value used to identify regression residuals outside the respective central portion of the distribution.
