# Supplementary material for: Genomic Analysis of Natural Selection and Phenotypic Variation in High-Altitude Mongolians
Source: PLoS Genet. 2013 Jul 18;9(7):e1003634. doi: 10.1371/journal.pgen.1003634 (PMC3715426; doi:10.1371/journal.pgen.1003634)
Supplement: Table S5 — Stepwise linear regression analysis of [Hb] and Tibetan high-altitude selection candidate gene haplotypes in 26 female DU Mongolians. (DOCX) [file pgen.1003634.s006.docx]

**Table S5. Stepwise linear regression analysis of [Hb] and Tibetan high-altitude selection candidate gene haplotypes in 26 female DU Mongolians**

| **Predictor** | **In Model** | **p** | **beta** |
| --- | --- | --- | --- |
| *Age* | 0 | 0.06 | 0.03 |
| *EGLN1* | 0 | 0.97 | -0.02 |
| *HIF1AN* | 0 | 0.65 | -0.23 |
| *NOS1* | 0 | 0.25 | 0.48 |
| *MCC* | 0 | 0.57 | -0.25 |
| *CYP17A1* | 0 | 0.14 | -0.85 |
| *EPAS1* | 0 | 1.00 | 0.00 |
| *HFE* | 0 | 0.99 | 0.00 |
| *HMOX2* | 0 | 0.53 | 0.33 |
| *PKLR* | 0 | 0.79 | 0.15 |
| *PPARA* | 0 | 0.53 | 0.38 |
| *PPARG* | 0 | 0.31 | -0.58 |
