# Supplementary material for: Genomic Analysis of Natural Selection and Phenotypic Variation in High-Altitude Mongolians
Source: PLoS Genet. 2013 Jul 18;9(7):e1003634. doi: 10.1371/journal.pgen.1003634 (PMC3715426; doi:10.1371/journal.pgen.1003634)
Supplement: Table S6 — Key variants for Tianjiao1 mtDNA and Y-chromosome haplogroup assignments. (DOCX) [file pgen.1003634.s007.docx]

**Table S6. Key variants for Tianjiao1 mtDNA and Y-chromosome haplogroup assignments**

| **Marker** | **Rs number** | **Position ^a^** | | **Variation ^b^** | | **Reference** | | **Mongolian** | **Haplogroup** | |
| --- | --- | --- | --- | --- | --- | --- | --- | --- | --- | --- |
| *mtDNA* |  |  | |  | |  | |  |  | |
| 769 | rs2853519 | 769 | | G->A | | G | | G | L3 | |
| 1018 | rs2856982 | 1018 | | A->G | | G | | G | L3 | |
| 10873 | rs2857284 | 10873 | | C->T | | T | | T | N | |
| 10398 | rs2853826 | 10398 | | A->G | | A | | A | N | |
| 12705 | rs2854122 | 12705 | | C->T | | C | | C | R | |
| 16223 | rs2853513 | 16223 | | C->T | | C | | C | R | |
| 73 | rs3087742 | 73 | | G->A | | A | | A | R0 | |
| 11719 | rs2853495 | 11719 | | A->G | | G | | G | R0 | |
| 14766 | rs3135031 | 14766 | | T->C | | C | | C | HV | |
| 2706 | rs2854128 | 2706 | | A->G | | A | | A | H | |
| 7028 | rs2015062 | 7028 | | C->T | | C | | C | H | |
| 13708 | rs28359178 | 13708 | | A->G | | G | | A | H18 | |
| 14364 | - | 14364 | | G->A | | G | | A | H18 | |
|  |  |  | |  | |  | |  |  | |
| *Y-chromosome* | |  | |  | |  | |  |  | |
| P69 | rs7892898 | | 14926420 | | G->A | | G | A | | P |
| P240 | rs6530605 | | 14598808 | | T->C | | T | C | | P |
| P244 | rs2740981 | | 14433100 | | G->A | | G | A | | P |
| Page83 | rs13305774 | | 14898094 | | A->G | | A | G | | P |
| M242 | rs8179021 | | 15018582 | | C->T | | C | T | | Q |
| P36.2 | - | | 14496441 | | G->T | | G | T | | Q1 |
| L232 | - | | 17516095 | | G->A | | G | A | | Q1 |
| L472 | rs35724598 | | 7014317 | | G->C | | G | C | | Q1a |
| L528 | - | | 18029008 | | T->C | | T | C | | Q1a3 |
| M120 | - | | 21907394 | | T->C | | T | C | | Q1a1 |
| M265 | rs3212294 | | 15030650 | | C->A | | C | A | | Q1a1 |

^a^ Position is relative to the revised Cambridge Reference Sequence (rCRS) and to GRCh37 for the mtDNA and Y-chromosome, respectively; ^b^ Ancestral allele -> derived allele.
