# Supplementary material for: Genomic Analysis of Natural Selection and Phenotypic Variation in High-Altitude Mongolians
Source: PLoS Genet. 2013 Jul 18;9(7):e1003634. doi: 10.1371/journal.pgen.1003634 (PMC3715426; doi:10.1371/journal.pgen.1003634)
Supplement: Table S7 — SIFT prediction for the effect of Tianjiao1-specific SNVs. (DOCX) [file pgen.1003634.s008.docx]

**Table S7. SIFT prediction for the effect of Tianjiao1-specific SNVs.**

| **Chr** | **Type** | **Start** | **End** | **Var Seq** | **Ref Seq** | **Effect** | **Gene** | **AA Sub** | **SIFT Prediction** |
| --- | --- | --- | --- | --- | --- | --- | --- | --- | --- |
| chr1 | SNV | 878184 | 878184 | T | C | Nonsynonymous | SAMD11 | P345S | TOLERATED |
| chr1 | SNV | 13182995 | 13182995 | C | G | Nonsynonymous | LOC440563 |  |  |
| chr1 | SNV | 16054156 | 16054156 | C | A | Nonsynonymous | PLEKHM2 | Q510P | TOLERATED |
| chr1 | SNV | 17409046 | 17409046 | A | C | Nonsynonymous | PADI2 | E384D | TOLERATED |
| chr1 | SNV | 21042129 | 21042129 | T | C | Nonsynonymous | KIF17 | V79I | DAMAGING |
| chr1 | SNV | 22924345 | 22924345 | A | G | Nonsynonymous | EPHA8 | V703I | TOLERATED |
| chr1 | SNV | 29138936 | 29138936 | T | C | Nonsynonymous | OPRD1 | P14L | TOLERATED |
| chr1 | SNV | 36556854 | 36556854 | G | A | Nonsynonymous | ADPRHL2 | Y74C | TOLERATED |
| chr1 | SNV | 38352592 | 38352592 | T | C | Nonsynonymous | INPP5B | E447K | TOLERATED |
| chr1 | SNV | 39340828 | 39340828 | T | C | Nonsynonymous | GJA9 | V315I | TOLERATED |
| chr1 | SNV | 41483638 | 41483638 | G | T | Nonsynonymous | LOC100507178 | K209T | TOLERATED |
| chr1 | SNV | 43108240 | 43108240 | G | A | Nonsynonymous | CCDC30 | I579V | TOLERATED |
| chr1 | SNV | 44458021 | 44458021 | C | G | Nonsynonymous | CCDC24 | L88F | TOLERATED |
| chr1 | SNV | 46976776 | 46976776 | A | G | Nonsynonymous | DMBX1 | S168N | TOLERATED |
| chr1 | SNV | 150955830 | 150955830 | T | C | Nonsynonymous | ANXA9 | T31I | TOLERATED |
| chr1 | SNV | 151259861 | 151259861 | C | T | Nonsynonymous | ZNF687 |  | Not scored |
| chr1 | SNV | 152191190 | 152191190 | C | T | Nonsynonymous | HRNR | Q972R | TOLERATED |
| chr1 | SNV | 158150923 | 158150923 | A | C | Nonsynonymous | CD1D | A11E | DAMAGING |
| chr1 | SNV | 158590141 | 158590141 | A | C | Nonsynonymous | SPTA1 | R2076L | TOLERATED |
| chr1 | SNV | 158590144 | 158590144 | T | A | Nonsynonymous | SPTA1 | I2075N | DAMAGING |
| chr1 | SNV | 160783582 | 160783582 | T | G | Nonsynonymous | LY9 | G204V | DAMAGING |
| chr1 | SNV | 161753858 | 161753858 | G | A | Nonsynonymous | ATF6 | D109G | TOLERATED |
| chr1 | SNV | 163325234 | 163325234 | A | G | Nonsynonymous | NUF2 | R457K | TOLERATED |
| chr1 | SNV | 165648771 | 165648771 | T | C | Nonsynonymous | ALDH9A1 | G280R | DAMAGING |
| chr1 | SNV | 172002248 | 172002248 | T | A | Nonsynonymous | DNM3 | Y231F | TOLERATED |
| chr1 | SNV | 205156703 | 205156703 | A | G | Nonsynonymous | DSTYK | A166V | TOLERATED |
| chr1 | SNV | 206652371 | 206652371 | A | G | Nonsynonymous | IKBKE | V360I | TOLERATED |
| chr1 | SNV | 207643193 | 207643193 | A | G | Nonsynonymous | CR2 | R324H | TOLERATED |
| chr1 | SNV | 208218070 | 208218070 | A | C | Nonsynonymous | PLXNA2 | M1219I | DAMAGING |
| chr1 | SNV | 213032071 | 213032071 | A | G | Nonsynonymous | FLVCR1 | A93T | TOLERATED |
| chr1 | SNV | 228540735 | 228540735 | A | C | Nonsynonymous | OBSCN | H6211Q | TOLERATED |
| chr1 | SNV | 229730829 | 229730829 | A | C | Nonsynonymous | TAF5L | D329Y | TOLERATED |
| chr1 | SNV | 235299029 | 235299029 | T | C | Nonsynonymous | RBM34 | V311M | DAMAGING |
| chr1 | SNV | 247076557 | 247076557 | C | T | Nonsynonymous | AHCTF1 | N187S | TOLERATED |
| chr10 | SNV | 85968116 | 85968116 | A | G | Nonsynonymous | CDHR1 | V384I | TOLERATED |
| chr10 | SNV | 85984866 | 85984866 | C | G | Nonsynonymous | LRIT2 | L39V | TOLERATED |
| chr10 | SNV | 87373271 | 87373271 | T | C | Nonsynonymous | GRID1 | A832T | DAMAGING |
| chr10 | SNV | 99342169 | 99342169 | C | G | Nonsynonymous | ANKRD2 | R278T | TOLERATED |
| chr10 | SNV | 104123091 | 104123091 | A | G | Nonsynonymous | GBF1 | R651Q | TOLERATED |
| chr10 | SNV | 105238645 | 105238645 | T | C | Nonsynonymous | CALHM3 | A49T | TOLERATED |
| chr10 | SNV | 133058581 | 133058581 | A | G | Nonsynonymous | TCERG1L | P266L | DAMAGING |
| chr10 | SNV | 135102420 | 135102420 | G | C | Nonsynonymous | TUBGCP2 | E489Q | TOLERATED |
| chr10 | SNV | 135195116 | 135195116 | G | A | Nonsynonymous | PAOX | D274G | DAMAGING |
| chr11 | SNV | 598457 | 598457 | T | C | Nonsynonymous | PHRF1 | R327C | DAMAGING |
| chr11 | SNV | 5255266 | 5255266 | C | A | Nonsynonymous | HBD | S90R | DAMAGING * |
| chr11 | SNV | 7982354 | 7982354 | A | G | Nonsynonymous | NLRP10 | P269S | TOLERATED |
| chr11 | SNV | 12316041 | 12316041 | A | G | Nonsynonymous | MICALCL | V355I | TOLERATED |
| chr11 | SNV | 18743487 | 18743487 | G | C | Nonsynonymous | IGSF22 | E71Q | TOLERATED |
| chr11 | SNV | 35338975 | 35338975 | C | G | Nonsynonymous | SLC1A2 | R36G | DAMAGING |
| chr11 | SNV | 44079938 | 44079938 | A | G | Nonsynonymous | ACCSL | E467K | TOLERATED |
| chr11 | SNV | 49207245 | 49207245 | A | G | Nonsynonymous | FOLH1 | L268F | DAMAGING |
| chr11 | SNV | 49229874 | 49229874 | T | C | Nonsynonymous | FOLH1 | G30S | TOLERATED |
| chr11 | SNV | 56000040 | 56000040 | G | A | Nonsynonymous | OR5T2 | C208R | DAMAGING |
| chr11 | SNV | 62299095 | 62299095 | T | G | Nonsynonymous | AHNAK | P932T | TOLERATED |
| chr11 | SNV | 62365580 | 62365580 | T | C | Nonsynonymous | MTA2 | V136M | TOLERATED |
| chr11 | MNP | 62417239 | 62417242 | AGGT | GACG | Nonsynonymous | INTS5 |  |  |
| chr11 | SNV | 66633792 | 66633792 | T | C | Nonsynonymous | PC | V351M | DAMAGING |
| chr11 | SNV | 67163780 | 67163780 | A | G | Nonsynonymous | RAD9A | V261I | TOLERATED |
| chr11 | SNV | 72421459 | 72421459 | T | C | Nonsynonymous | ARAP1 | D218N | TOLERATED |
| chr11 | SNV | 93469364 | 93469364 | G | T | Nonsynonymous | TAF1D | K267T | TOLERATED |
| chr11 | SNV | 119183298 | 119183298 | T | C | Nonsynonymous | MCAM | R267H | TOLERATED |
| chr12 | SNV | 980497 | 980497 | G | A | Nonsynonymous | WNK1 | I736V | TOLERATED |
| chr12 | SNV | 995173 | 995173 | A | T | Nonsynonymous | WNK1 | S908T | TOLERATED |
| chr12 | SNV | 8672931 | 8672931 | A | G | Nonsynonymous | CLEC4D |  | Not scored |
| chr12 | SNV | 10978472 | 10978472 | C | T | Nonsynonymous | TAS2R10 | I133V | TOLERATED |
| chr12 | SNV | 25257327 | 25257327 | A | G | Nonsynonymous | LRMP | R360H | DAMAGING |
| chr12 | SNV | 64823860 | 64823860 | T | A | Nonsynonymous | XPOT |  | Not scored |
| chr12 | SNV | 72024726 | 72024726 | T | G | Nonsynonymous | ZFC3H1 | P1160Q | DAMAGING |
| chr12 | SNV | 111748390 | 111748390 | G | A | Nonsynonymous | CUX2 | T602A | DAMAGING |
| chr12 | SNV | 111923534 | 111923534 | G | C | Nonsynonymous | ATXN2 | V974L | TOLERATED |
| chr12 | SNV | 113549898 | 113549898 | G | T | Nonsynonymous | RASAL1 | E456A | TOLERATED |
| chr12 | SNV | 114386754 | 114386754 | T | C | Nonsynonymous | RBM19 | R387Q | TOLERATED |
| chr12 | SNV | 123107112 | 123107112 | G | A | Nonsynonymous | KNTC1 | N2158S | TOLERATED |
| chr12 | SNV | 128899835 | 128899835 | G | A | Nonsynonymous | TMEM132C | K215R | TOLERATED |
| chr12 | SNV | 132512674 | 132512674 | A | T | Nonsynonymous | EP400 | L1660Q | TOLERATED |
| chr13 | SNV | 35685074 | 35685074 | C | T | Nonsynonymous | NBEA | I654T | TOLERATED |
| chr13 | SNV | 46656622 | 46656622 | A | G | Nonsynonymous | CPB2 | P113L | TOLERATED |
| chr13 | SNV | 96555164 | 96555164 | T | C | Nonsynonymous | UGGT2 | E816K | TOLERATED |
| chr13 | SNV | 109793571 | 109793571 | G | A | Nonsynonymous | MYO16 |  | Not scored |
| chr14 | SNV | 21899028 | 21899028 | T | C | Nonsynonymous | CHD8 | A259T | TOLERATED |
| chr14 | SNV | 23885038 | 23885038 | C | T | Nonsynonymous | MYH7 | T1653A | TOLERATED |
| chr14 | SNV | 24538140 | 24538140 | A | G | Nonsynonymous | LRRC16B | S369N | TOLERATED |
| chr14 | SNV | 24615789 | 24615789 | G | A | Nonsynonymous | PSME2 | M1T | DAMAGING |
| chr14 | SNV | 24883915 | 24883915 | T | C | Nonsynonymous | NYNRIN | P987L | DAMAGING * |
| chr14 | SNV | 59988313 | 59988313 | A | G | Nonsynonymous | C14orf38 | R693W | TOLERATED |
| chr14 | SNV | 60581449 | 60581449 | G | A | Nonsynonymous | C14orf135 | M7V | TOLERATED |
| chr14 | SNV | 62014512 | 62014512 | A | G | Nonsynonymous | PRKCH | E605K | DAMAGING |
| chr14 | SNV | 65519973 | 65519973 | G | A | Nonsynonymous | FNTB | M325V | TOLERATED |
| chr14 | SNV | 102467378 | 102467378 | G | A | Nonsynonymous | DYNC1H1 | R1388G | TOLERATED |
| chr14 | SNV | 105413494 | 105413494 | A | G | Nonsynonymous | AHNAK2 | A2765V | TOLERATED |
| chr14 | MNP | 105413516 | 105413517 | GC | CG | Nonsynonymous | AHNAK2 |  |  |
| chr14 | SNV | 105416270 | 105416270 | C | T | Nonsynonymous | AHNAK2 | K1840E | TOLERATED |
| chr14 | MNP | 105416384 | 105416385 | CA | TG | Nonsynonymous | AHNAK2 |  |  |
| chr14 | SNV | 105416505 | 105416505 | T | C | Nonsynonymous | AHNAK2 | M1761I | TOLERATED |
| chr14 | SNV | 105416541 | 105416541 | G | C | Nonsynonymous | AHNAK2 | L1749F | TOLERATED |
| chr15 | SNV | 22368810 | 22368810 | G | C | Nonsynonymous | LOC727924 | P79A | DAMAGING |
| chr15 | SNV | 22743326 | 22743326 | A | G | Nonsynonymous | GOLGA6L1 | E571K | TOLERATED |
| chr15 | SNV | 22743347 | 22743347 | A | G | Nonsynonymous | GOLGA6L1 | E578K | TOLERATED |
| chr15 | SNV | 40462304 | 40462304 | G | C | Nonsynonymous | BUB1B | P74R | DAMAGING |
| chr15 | SNV | 40632155 | 40632155 | T | C | Nonsynonymous | C15orf52 | R69K | TOLERATED |
| chr15 | SNV | 42133075 | 42133075 | A | G | Nonsynonymous | JMJD7 | R339Q | TOLERATED |
| chr15 | SNV | 48717652 | 48717652 | C | T | Nonsynonymous | FBN1 | N2456S | TOLERATED |
| chr16 | SNV | 847859 | 847859 | T | G | Nonsynonymous | CHTF18 | D938Y | DAMAGING |
| chr16 | SNV | 3022997 | 3022997 | A | C | Nonsynonymous | PAQR4 | R486L | DAMAGING |
| chr16 | SNV | 3070438 | 3070438 | T | G | Nonsynonymous | TNFRSF12A | V14L | TOLERATED |
| chr16 | SNV | 12996291 | 12996291 | C | G | Nonsynonymous | SHISA9 | D165H | DAMAGING |
| chr16 | SNV | 22360643 | 22360643 | T | C | Nonsynonymous | CDR2 | A155T | TOLERATED |
| chr16 | SNV | 68398925 | 68398925 | A | G | Nonsynonymous | SMPD3 | P465L | TOLERATED |
| chr16 | SNV | 69752348 | 69752348 | C | T | Nonsynonymous | NQO1 |  | Not scored |
| chr16 | SNV | 88804443 | 88804443 | T | C | Nonsynonymous | FAM38A | G307S | TOLERATED |
| chr17 | SNV | 263145 | 263145 | T | C | Nonsynonymous | C17orf97 | R171S | TOLERATED |
| chr17 | SNV | 4349487 | 4349487 | A | G | Nonsynonymous | SPNS3 | V56I | DAMAGING |
| chr17 | SNV | 7246852 | 7246852 | T | C | Nonsynonymous | ACAP1 | R167W | DAMAGING |
| chr17 | SNV | 8296520 | 8296520 | A | G | Nonsynonymous | RNF222 | T87M | TOLERATED |
| chr17 | SNV | 15142830 | 15142830 | G | C | Nonsynonymous | PMP22 | G93R | DAMAGING |
| chr17 | SNV | 17106166 | 17106166 | T | C | Nonsynonymous | PLD6 | S225N | DAMAGING * |
| chr17 | SNV | 39913725 | 39913725 | T | C | Nonsynonymous | JUP | R663H | DAMAGING * |
| chr17 | SNV | 42988801 | 42988801 | T | C | Nonsynonymous | GFAP | M310I | TOLERATED |
| chr17 | SNV | 48753779 | 48753779 | T | A | Nonsynonymous | ABCC3 | N1070Y | DAMAGING |
| chr17 | SNV | 56277147 | 56277147 | C | T | Nonsynonymous | EPX | V510A | DAMAGING |
| chr17 | SNV | 56544322 | 56544322 | A | G | Nonsynonymous | HSF5 | S315F | DAMAGING |
| chr17 | SNV | 58121201 | 58121201 | A | C | Nonsynonymous | HEATR6 | C1090F | TOLERATED |
| chr17 | SNV | 58303550 | 58303550 | T | C | Nonsynonymous | USP32 | G428R | TOLERATED |
| chr17 | SNV | 72954840 | 72954840 | T | G | Nonsynonymous | C17orf28 | L162M | DAMAGING |
| chr17 | SNV | 74383132 | 74383132 | A | G | Nonsynonymous | SPHK1 | R293H | TOLERATED |
| chr18 | SNV | 3134744 | 3134744 | C | T | Nonsynonymous | MYOM1 | K763R | TOLERATED |
| chr18 | SNV | 29339873 | 29339873 | A | G | Nonsynonymous | MCART2 | P261L | TOLERATED |
| chr18 | SNV | 33736484 | 33736484 | C | T | Nonsynonymous | ELP2 | L439S | DAMAGING |
| chr18 | SNV | 44559927 | 44559927 | A | C | Nonsynonymous | KATNAL2 | R570I | TOLERATED |
| chr19 | SNV | 4847711 | 4847711 | C | G | Nonsynonymous | PLIN3 | L276V | TOLERATED |
| chr19 | SNV | 7696633 | 7696633 | A | G | Nonsynonymous | PCP2 | P118L | TOLERATED |
| chr19 | SNV | 8615202 | 8615202 | T | C | Nonsynonymous | MYO1F | G360R | TOLERATED |
| chr19 | SNV | 9048845 | 9048845 | T | C | Nonsynonymous | MUC16 | G10929E | Not Predicted |
| chr19 | SNV | 14273649 | 14273649 | T | C | Nonsynonymous | LPHN1 | V327I | DAMAGING * |
| chr19 | SNV | 22156512 | 22156512 | G | C | Nonsynonymous | ZNF208 | E442Q | TOLERATED |
| chr19 | SNV | 35837547 | 35837547 | A | G | Nonsynonymous | CD22 | G831R | TOLERATED |
| chr19 | SNV | 36350384 | 36350384 | A | C | Nonsynonymous | KIRREL2 | T175N | TOLERATED |
| chr19 | SNV | 38102446 | 38102446 | C | T | Nonsynonymous | ZNF540 | S89P | TOLERATED |
| chr19 | SNV | 39597600 | 39597600 | A | G | Nonsynonymous | PAPL | R376Q | TOLERATED |
| chr19 | SNV | 40354519 | 40354519 | T | C | Nonsynonymous | FCGBP | R5317Q | TOLERATED |
| chr19 | SNV | 41355733 | 41355733 | T | G | Nonsynonymous | CYP2A6 | F111L | TOLERATED |
| chr19 | SNV | 44006363 | 44006363 | T | C | Nonsynonymous | PHLDB3 | G96R | TOLERATED |
| chr19 | SNV | 44417682 | 44417682 | T | C | Nonsynonymous | ZNF45 | G636R | DAMAGING |
| chr19 | SNV | 47920111 | 47920111 | T | C | Nonsynonymous | MEIS3 | V99I | DAMAGING |
| chr19 | SNV | 51650550 | 51650550 | G | C | Nonsynonymous | SIGLEC7 | N306K | TOLERATED |
| chr19 | SNV | 52034159 | 52034159 | A | G | Nonsynonymous | SIGLEC6 | P161L | TOLERATED |
| chr19 | SNV | 52034175 | 52034175 | C | G | Nonsynonymous | SIGLEC6 | L156V | TOLERATED |
| chr19 | SNV | 55401040 | 55401040 | A | G | Nonsynonymous | FCAR | T129T | TOLERATED |
| chr19 | SNV | 55738797 | 55738797 | A | G | Nonsynonymous | TMEM86B | P145S | TOLERATED |
| chr19 | SNV | 57060489 | 57060489 | T | C | Nonsynonymous | ZFP28 | P229L | TOLERATED |
| chr2 | SNV | 10717763 | 10717763 | G | C | Nonsynonymous | NOL10 | R599T | TOLERATED |
| chr2 | SNV | 21230333 | 21230333 | T | C | Nonsynonymous | APOB | R3136H | TOLERATED |
| chr2 | SNV | 24516570 | 24516570 | C | G | Nonsynonymous | ITSN2 | F570L | TOLERATED |
| chr2 | MNP | 26537362 | 26537363 | CA | GG | Nonsynonymous | GPR113 |  |  |
| chr2 | SNV | 29274645 | 29274645 | T | C | Nonsynonymous | FAM179A | R916W | TOLERATED |
| chr2 | SNV | 73868638 | 73868638 | A | G | Nonsynonymous | NAT8 | P40S | TOLERATED |
| chr2 | SNV | 113326338 | 113326338 | T | G | Nonsynonymous | POLR1B | A645S | TOLERATED |
| chr2 | SNV | 160807986 | 160807986 | A | C | Nonsynonymous | PLA2R1 | W1135C | DAMAGING |
| chr2 | SNV | 171319953 | 171319953 | T | C | Nonsynonymous | MYO3B | R935W | DAMAGING |
| chr2 | SNV | 189599490 | 189599490 | A | G | Nonsynonymous | DIRC1 | A53V | Not Predicted |
| chr2 | SNV | 216240027 | 216240027 | T | C | Nonsynonymous | FN1 | G1933S | DAMAGING |
| chr2 | SNV | 217234673 | 217234673 | T | A | Nonsynonymous | MARCH4 | V104E | TOLERATED |
| chr2 | SNV | 223423385 | 223423385 | C | T | Nonsynonymous | SGPP2 | L323S | TOLERATED |
| chr2 | SNV | 224746659 | 224746659 | C | T | Nonsynonymous | WDFY1 |  |  |
| chr2 | SNV | 242312656 | 242312656 | G | A | Nonsynonymous | FARP2 | H45R | TOLERATED |
| chr20 | SNV | 1293237 | 1293237 | T | C | Nonsynonymous | FKBP1A | R185Q | DAMAGING |
| chr20 | SNV | 2638855 | 2638855 | T | C | Nonsynonymous | NOP56 | P567L | DAMAGING * |
| chr20 | SNV | 5093667 | 5093667 | C | G | Nonsynonymous | C20orf30 | P3R | DAMAGING * |
| chr20 | SNV | 37580397 | 37580397 | A | G | Nonsynonymous | FAM83D | R361Q | TOLERATED |
| chr20 | SNV | 55206895 | 55206895 | G | A | Nonsynonymous | TFAP2C | Q190R | TOLERATED |
| chr20 | SNV | 62737344 | 62737344 | G | C | Nonsynonymous | NPBWR2 | V281L | TOLERATED |
| chr22 | SNV | 30403188 | 30403188 | G | C | Nonsynonymous | MTMR3 | L117V | DAMAGING |
| chr22 | SNV | 38051336 | 38051336 | A | G | Nonsynonymous | SH3BP1 |  | Not scored |
| chr22 | SNV | 44064859 | 44064859 | C | T | Nonsynonymous | EFCAB6 | I569M | TOLERATED |
| chr3 | SNV | 9754227 | 9754227 | C | G | Nonsynonymous | CPNE9 | G127A | TOLERATED |
| chr3 | SNV | 10452482 | 10452482 | C | G | Nonsynonymous | ATP2B2 | P73A | TOLERATED |
| chr3 | SNV | 11421450 | 11421450 | C | G | Nonsynonymous | ATG7 | R627P | DAMAGING |
| chr3 | SNV | 39178730 | 39178730 | A | C | Nonsynonymous | TTC21A | A1109D | TOLERATED |
| chr3 | MNP | 44328999 | 44329000 | AT | GA | Nonsynonymous | C3orf77 |  |  |
| chr3 | SNV | 47742774 | 47742774 | G | T | Nonsynonymous | SMARCC1 | K387Q | TOLERATED |
| chr3 | SNV | 75787273 | 75787273 | T | G | Nonsynonymous | ZNF717 | H494N | DAMAGING |
| chr3 | SNV | 87325581 | 87325581 | T | G | Nonsynonymous | POU1F1 | T11N | TOLERATED |
| chr3 | SNV | 97869037 | 97869037 | G | C | Nonsynonymous | OR5H14 | Q270E | TOLERATED |
| chr3 | SNV | 108298489 | 108298489 | C | T | Nonsynonymous | KIAA1524 | H199R | DAMAGING |
| chr3 | SNV | 108363395 | 108363395 | A | G | Nonsynonymous | DZIP3 | R509K | TOLERATED |
| chr3 | SNV | 121575926 | 121575926 | A | G | Nonsynonymous | EAF2 | R136K | TOLERATED |
| chr3 | SNV | 128603562 | 128603562 | T | C | Nonsynonymous | ACAD9 | P73S | TOLERATED |
| chr3 | SNV | 140997267 | 140997267 | G | C | Nonsynonymous | ACPL2 | P55A | TOLERATED |
| chr3 | SNV | 145841930 | 145841930 | T | C | Nonsynonymous | PLOD2 | V66M | DAMAGING |
| chr3 | SNV | 149469216 | 149469216 | A | G | Nonsynonymous | COMMD2 | L68F | DAMAGING |
| chr3 | SNV | 154056041 | 154056041 | A | G | Nonsynonymous | GPR149 | A548V | DAMAGING * |
| chr3 | SNV | 160143869 | 160143869 | C | T | Nonsynonymous | SMC4 | I829T | TOLERATED |
| chr3 | SNV | 168802737 | 168802737 | A | G | Nonsynonymous | MECOM | A1104V | DAMAGING |
| chr4 | SNV | 1657045 | 1657045 | A | G | Nonsynonymous | FAM53A | S181L | DAMAGING |
| chr4 | SNV | 2073872 | 2073872 | A | G | Nonsynonymous | HAUS3 | P572S | TOLERATED |
| chr4 | SNV | 2460932 | 2460932 | T | C | Nonsynonymous | LOC402160 | R213C | DAMAGING |
| chr4 | SNV | 5843059 | 5843059 | C | T | Nonsynonymous | CRMP1 | I377V | TOLERATED |
| chr4 | SNV | 7780516 | 7780516 | T | C | Nonsynonymous | AFAP1 | A540T | TOLERATED |
| chr4 | SNV | 25379167 | 25379167 | G | A | Nonsynonymous | ANAPC4 | T40A | TOLERATED |
| chr4 | SNV | 55141095 | 55141095 | T | C | Nonsynonymous | PDGFRA | P341S | DAMAGING * |
| chr4 | SNV | 75937929 | 75937929 | A | G | Nonsynonymous | PARM1 | S113N | DAMAGING |
| chr4 | SNV | 85687053 | 85687053 | C | T | Nonsynonymous | WDFY3 | I1700V | TOLERATED |
| chr4 | SNV | 102984248 | 102984248 | G | T | Nonsynonymous | BANK1 | L722R | DAMAGING |
| chr4 | SNV | 129873940 | 129873940 | G | A | Nonsynonymous | SCLT1 |  | Not scored |
| chr4 | SNV | 153691825 | 153691825 | T | C | Nonsynonymous | TIGD4 | R111H | TOLERATED |
| chr4 | SNV | 187078781 | 187078781 | A | C | Nonsynonymous | FAM149A | P213T | TOLERATED |
| chr5 | SNV | 14713075 | 14713075 | T | C | Nonsynonymous | ANKH | G425S | DAMAGING |
| chr5 | SNV | 56777648 | 56777648 | A | G | Nonsynonymous | ACTBL2 | A296V | Not Predicted |
| chr5 | SNV | 74096792 | 74096792 | A | C | Nonsynonymous | FAM169A | G339V | TOLERATED |
| chr5 | SNV | 111576498 | 111576498 | C | T | Nonsynonymous | EPB41L4A | T269A | DAMAGING |
| chr5 | SNV | 115177216 | 115177216 | T | G | Nonsynonymous | ATG12 | P59T | DAMAGING |
| chr5 | SNV | 115783100 | 115783100 | A | G | Nonsynonymous | SEMA6A | R785W | DAMAGING |
| chr5 | SNV | 130928107 | 130928107 | T | C | Nonsynonymous | RAPGEF6 | V134M | TOLERATED |
| chr5 | SNV | 140347339 | 140347339 | G | T | Nonsynonymous | PCDHAC1 | S330A | TOLERATED |
| chr5 | SNV | 169689733 | 169689733 | T | G | Nonsynonymous | LCP2 |  | Not scored |
| chr6 | SNV | 5187181 | 5187181 | C | G | Nonsynonymous | LYRM4 | H81D | TOLERATED |
| chr6 | SNV | 26413895 | 26413895 | T | C | Nonsynonymous | BTN3A1 | T506M | DAMAGING |
| chr6 | SNV | 29142182 | 29142182 | C | T | Nonsynonymous | OR2J2_DUP_07 | M257T | DAMAGING |
| chr6 | SNV | 30954338 | 30954338 | T | C | Nonsynonymous | MUC21_DUP_06 |  | Not scored |
| chr6 | SNV | 32064893 | 32064893 | A | T | Nonsynonymous | TNXB_DUP_10 | Q246L | TOLERATED |
| chr6 | SNV | 76063261 | 76063261 | T | C | Nonsynonymous | FILIP1 | R208Q | TOLERATED |
| chr6 | SNV | 83900563 | 83900563 | C | T | Nonsynonymous | PGM3 | I85V | TOLERATED |
| chr6 | SNV | 93967964 | 93967964 | C | G | Nonsynonymous | EPHA7 | P655A | DAMAGING |
| chr6 | SNV | 129634117 | 129634117 | T | G | Nonsynonymous | LAMA2 | G1096C | DAMAGING |
| chr6 | SNV | 159173006 | 159173006 | T | C | Nonsynonymous | SYTL3 | R361C | DAMAGING |
| chr6 | SNV | 159178375 | 159178375 | G | A | Nonsynonymous | SYTL3 | T424A | TOLERATED |
| chr6 | SNV | 168315362 | 168315362 | G | A | Nonsynonymous | MLLT4 | Y741C | TOLERATED |
| chr7 | SNV | 4839385 | 4839385 | T | C | Nonsynonymous | RADIL | A971T | TOLERATED |
| chr7 | SNV | 21469852 | 21469852 | T | C | Nonsynonymous | SP4 | R357C | DAMAGING |
| chr7 | SNV | 27147898 | 27147898 | T | G | Nonsynonymous | HOXA3 | P323Q | DAMAGING |
| chr7 | SNV | 27147926 | 27147926 | G | C | Nonsynonymous | HOXA3 | A314P | TOLERATED |
| chr7 | SNV | 42005922 | 42005922 | C | T | Nonsynonymous | GLI3 | S917G | TOLERATED |
| chr7 | SNV | 47409150 | 47409150 | A | G | Nonsynonymous | TNS3 | P365S | TOLERATED |
| chr7 | SNV | 51152907 | 51152907 | A | G | Nonsynonymous | COBL | P351L | DAMAGING |
| chr7 | SNV | 63679749 | 63679749 | C | T | Nonsynonymous | ZNF735 |  |  |
| chr7 | SNV | 70242155 | 70242155 | A | G | Nonsynonymous | AUTS2 |  | Not scored |
| chr7 | SNV | 87174186 | 87174186 | A | G | Nonsynonymous | ABCB1 | R673C | TOLERATED |
| chr7 | SNV | 99753020 | 99753020 | C | G | Nonsynonymous | C7orf43 | P104R | DAMAGING * |
| chr7 | SNV | 100470912 | 100470912 | T | C | Nonsynonymous | TRIP6 | T473I | DAMAGING |
| chr7 | SNV | 100731797 | 100731797 | G | A | Nonsynonymous | TRIM56 | T402A | TOLERATED |
| chr7 | SNV | 107720227 | 107720227 | A | G | Nonsynonymous | LAMB4 | S569L | TOLERATED |
| chr7 | SNV | 142563850 | 142563850 | T | G | Nonsynonymous | EPHB6 | R413L | TOLERATED |
| chr7 | SNV | 156976575 | 156976575 | A | C | Nonsynonymous | UBE3C | A332D | TOLERATED |
| chr8 | SNV | 27145141 | 27145141 | A | G | Nonsynonymous | TRIM35 | R470W | TOLERATED |
| chr8 | SNV | 59410841 | 59410841 | G | A | Nonsynonymous | CYP7A1 | C90R | TOLERATED |
| chr8 | SNV | 88886001 | 88886001 | G | A | Nonsynonymous | DCAF4L2 | S67P | DAMAGING |
| chr9 | SNV | 35711280 | 35711280 | C | G | Nonsynonymous | TLN1 | L1331V | TOLERATED |
| chr9 | SNV | 114341165 | 114341165 | G | C | Nonsynonymous | PTGR1 | D65H | DAMAGING |
| chr9 | SNV | 131469059 | 131469059 | A | G | Nonsynonymous | PKN3 | R160Q | DAMAGING |
| chr9 | SNV | 139752710 | 139752710 | A | G | Nonsynonymous | MAMDC4 | G886R | TOLERATED |
| chr9 | SNV | 139960719 | 139960719 | T | C | Nonsynonymous | C9orf140 | G227S | TOLERATED |
| chr9 | SNV | 141015292 | 141015292 | G | A | Nonsynonymous | CACNA1B | T1344A | TOLERATED |
| chrX | SNV | 35966493 | 35966493 | A | G | Nonsynonymous | CXorf22 | V194M | DAMAGING |
| chrX | SNV | 49963354 | 49963354 | G | A | Nonsynonymous | AKAP4 |  | Not scored |
| chrX | SNV | 150869437 | 150869437 | C | G | Nonsynonymous | PRRG3 |  | Not scored |
| chr1 | SNV | 152283380 | 152283380 | A | G | Stop gained | FLG | Q1328* | N/A |
| chr10 | SNV | 74994624 | 74994624 | T | C | Stop gained | FAM149B1 | R468* | N/A |
| chr19 | SNV | 48789663 | 48789663 | G | C | Stop gained | ZNF114 | S261* | N/A |
| chr6 | SNV | 97597763 | 97597763 | A | G | Stop gained | MIR548H3 | Q1206* | N/A |
| chr9 | SNV | 103275606 | 103275606 | T | G | Stop gained | TMEFF1 | G113* | N/A |
| chr5 | SNV | 148697504 | 148697504 | T | G | Splice donor | AFAP1L1 |  |  |
| chr12 | SNV | 102542007 | 102542007 | A | G | Splice acceptor | C12orf48 |  |  |
| chr9 | SNV | 127662827 | 127662827 | A | C | Splice acceptor | GOLGA1 |  |  |

DAMAGING *: Low confidence prediction.
