# Supplementary material for: Genomic Analysis of Natural Selection and Phenotypic Variation in High-Altitude Mongolians
Source: PLoS Genet. 2013 Jul 18;9(7):e1003634. doi: 10.1371/journal.pgen.1003634 (PMC3715426; doi:10.1371/journal.pgen.1003634)
Supplement: Table S8 — SIFT prediction for the effect of Tianjiao1-specific Indels. (DOCX) [file pgen.1003634.s009.docx]

**Table S8. SIFT prediction for the effect of Tianjiao1-specific Indels.**

| **Chr** | **Type** | **Start** | **End** | **Var Seq** | **Ref Seq** | **Effect** | **Gene** | **NMD^a^** | **Indel location** |
| --- | --- | --- | --- | --- | --- | --- | --- | --- | --- |
| chr1 | nucleotide_deletion | 62579839 | 62579841 | - | GAG | inframe | INADL | NO | 98% |
| chr10 | nucleotide_insertion | 124921830 | 124921830 | AAG | - | inframe | BUB3 | YES | 67% |
| chr11 | complex_substitution | 309147 | 309148 | GGTCC | CG | inframe | IFITM2 |  |  |
| chr11 | nucleotide_insertion | 64337176 | 64337176 | ATG | - | inframe | SLC22A11 | NO | 87% |
| chr12 | nucleotide_deletion | 118506328 | 118506333 | - | TCCTCC | inframe | VSIG10 | YES | 87% |
| chr15 | nucleotide_insertion | 49284783 | 49284783 | TTC | - | inframe | SECISBP2L | NO | 89% |
| chr18 | nucleotide_deletion | 28649002 | 28649004 | - | CCT | inframe | DSC2 | NO | 87% |
| chr19 | nucleotide_insertion | 36002422 | 36002422 | CTGCTGCTG | - | inframe | DMKN | NO | 57% |
| chr2 | nucleotide_insertion | 133403084 | 133403084 | AAG | - | inframe | GPR39 | NO | 93% |
| chr3 | nucleotide_deletion | 46751086 | 46751088 | - | AAG | inframe | TMIE | NO | 80% |
| chr4 | nucleotide_deletion | 3494840 | 3494842 | - | CAG | inframe | DOK7 | NO | 74% |
| chr4 | nucleotide_deletion | 42145560 | 42145562 | - | CTC | inframe | BEND4 | YES | 45% |
| chr4 | nucleotide_deletion | 88536883 | 88536900 | - | TAGCAGTGACAGCAGCAA | inframe | DSPP | NO | 79% |
| chr9 | nucleotide_insertion | 100092982 | 100092982 | AGGAGG | - | inframe | C9orf174 | NO | 56% |
| chr9 | nucleotide_deletion | 125618125 | 125618127 | - | TTC | inframe | RC3H2 | YES | 70% |
| chr9 | nucleotide_deletion | 134019968 | 134019973 | - | CACCCC | inframe | NUP214 | YES | 25% |
| chr9 | nucleotide_deletion | 135203945 | 135203947 | - | TAA | inframe | SETX | YES | 38% |
| chr1 | nucleotide_insertion | 90178768 | 90178768 | A | - | frameshift | LRRC8C | NO | 26% |
| chr1 | nucleotide_insertion | 182812483 | 182812483 | A | - | frameshift | DHX9 | YES | 4% |
| chr1 | nucleotide_deletion | 196928052 | 196928053 | - | AT | frameshift | CFHR2 | NO | 80% |
| chr11 | nucleotide_insertion | 4567095 | 4567095 | T | - | frameshift | OR52M1 | N/A | 71% |
| chr11 | nucleotide_insertion | 67412231 | 67412231 | AC | - | frameshift | ACY3 | NO | 0% |
| chr12 | nucleotide_deletion | 57005751 | 57005752 | - | GA | frameshift | BAZ2A | YES | 24% |
| chr15 | nucleotide_insertion | 41795056 | 41795056 | C | - | frameshift | ITPKA | NO | 85% |
| chr15 | nucleotide_insertion | 65627742 | 65627742 | C | - | frameshift | IGDCC3 | YES | 23% |
| chr16 | nucleotide_insertion | 68331210 | 68331210 | A | - | frameshift | SLC7A6 | NO | 99% |
| chr16 | nucleotide_deletion | 81399024 | 81399024 | - | C | frameshift | GAN | YES | 80% |
| chr16 | nucleotide_insertion | 88495412 | 88495412 | C | - | frameshift | ZNF469 | YES | 13% |
| chr17 | nucleotide_insertion | 29548924 | 29548924 | C | - | frameshift | NF1 | YES | 20% |
| chr17 | nucleotide_insertion | 39684407 | 39684407 | A | - | frameshift | KRT19 | YES | 8% |
| chr17 | nucleotide_deletion | 39684411 | 39684411 | - | G | frameshift | KRT19 | YES | 7% |
| chr17 | nucleotide_deletion | 73999342 | 73999343 | - | CT | frameshift | C17orf106 | NO | 71% |
| chr19 | nucleotide_insertion | 20728108 | 20728108 | T | - | frameshift | ZNF737 | NO | 56% |
| chr2 | nucleotide_insertion | 24207579 | 24207579 | C | - | frameshift | UBXN2A | YES | 59% |
| chr2 | nucleotide_insertion | 27717525 | 27717525 | G | - | frameshift | FNDC4 | NO | 3% |
| chr2 | nucleotide_insertion | 162891786 | 162891786 | A | - | frameshift | DPP4 | YES | 29% |
| chr2 | nucleotide_deletion | 191381083 | 191381083 | - | C | frameshift | TMEM194B | YES | 51% |
| chr3 | nucleotide_deletion | 32576045 | 32576046 | - | TA | frameshift | DYNC1LI1 | YES | 59% |
| chr3 | nucleotide_insertion | 48207343 | 48207343 | T | - | frameshift | CDC25A | YES | 68% |
| chr3 | nucleotide_insertion | 52454441 | 52454441 | T | - | frameshift | PHF7 | YES | 35% |
| chr3 | nucleotide_deletion | 97887850 | 97887850 | - | T | frameshift | OR5H15 | N/A | 33% |
| chr4 | nucleotide_insertion | 15688772 | 15688772 | A | - | frameshift | FAM200B | N/A | 9% |
| chr4 | nucleotide_deletion | 17812767 | 17812767 | - | A | frameshift | NCAPG | YES | 2% |
| chr4 | nucleotide_insertion | 55987332 | 55987332 | G | - | frameshift | KDR | YES | 2% |
| chr4 | nucleotide_insertion | 128819613 | 128819613 | A | - | frameshift | PLK4 | NO | 97% |
| chr4 | nucleotide_insertion | 128842780 | 128842780 | G | - | frameshift | MFSD8 | NO | 80% |
| chr7 | nucleotide_insertion | 15405146 | 15405146 | A | - | frameshift | AGMO | NO | 94% |
| chr7 | nucleotide_deletion | 70255592 | 70255593 | - | CC | frameshift | AUTS2 | NO | 90% |
| chr8 | nucleotide_insertion | 89054014 | 89054014 | A | - | frameshift | MMP16 | NO | 82% |
| chr9 | nucleotide_deletion | 5231638 | 5231639 | - | TT | frameshift | INSL4 | NO | 27% |
| chr9 | nucleotide_insertion | 131287687 | 131287687 | G | - | frameshift | GLE1 | YES | 53% |
| chr9 | nucleotide_insertion | 114521435 | 114521435 | A | - | splice donor | C9orf84 |  |  |
| chr6 | nucleotide_insertion | 42611951 | 42611951 | A | - | splice acceptor | UBR2 |  |  |

^a^ nonsense-mediated decay
